# Spatiotemporal hierarchy in antibody recognition against transmitted HIV-1 envelope glycoprotein during natural infection

**DOI:** 10.1186/s12977-016-0243-3

**Published:** 2016-02-17

**Authors:** Su Jin, Yangtao Ji, Qian Wang, Hua Wang, Xuanling Shi, Xiaoxu Han, Tongqing Zhou, Hong Shang, Linqi Zhang

**Affiliations:** Comprehensive AIDS Research Center, Collaborative Innovation Center for Diagnosis and Treatment of Infectious Diseases, School of Life Sciences and School of Medicine, Tsinghua University, Beijing, 100084 China; Key Laboratory of AIDS Immunology of Ministry of Health, Department of Laboratory Medicine, No. 1 Hospital of China Medical University, Shenyang, 110001 China; Vaccine Research Center, National Institute of Allergy and Infectious Diseases, National Institutes of Health, Bethesda, MD 20892 USA

**Keywords:** HIV, Transmission, Antibody, Vaccine

## Abstract

**Background:**

Majority of HIV-1 infection is established by one transmitted/founder virus and understanding how the neutralizing antibodies develop against this virus is critical for our rational design an HIV-1 vaccine.

**Results:**

We report here antibody profiling of sequential plasma samples against transmitted/founder HIV-1 envelope glycoprotein in an epidemiologically linked transmission pair using our previously reported antigen library approach. We have decomposed the antibody recognition into three major subdomains on the envelope and showed their development in vivo followed a spatiotemporal hierarchy: starting with the ectodomain of gp41 at membrane proximal region, then the V3C3V4 and the V1V2 of gp120 at the membrane distal region. While antibodies to these subdomains appeared to undergo avidity maturation, the early anti-gp41 antibodies failed to translate into detectable autologous neutralization. Instead, it was the much delayed anti-V3C3V4 and anti-V1V2 antibodies constituted the major neutralizing activities.

**Conclusions:**

Our results indicate that the initial antibody response was severely misguided by the transmitted/founder virus and future vaccine design would need to avoid the ectodomain of gp41 and focus on the neutralizing targets in the V3C3V4 and V1V2 subdomains of gp120.

**Electronic supplementary material:**

The online version of this article (doi:10.1186/s12977-016-0243-3) contains supplementary material, which is available to authorized users.

## Background

Neutralizing antibodies are the major component of protective immunity against viral infection in humans. Polyclonal by nature, they exert their function by targeting the crucial antigenic domains on the viral envelop glycoprotein. Identifying the neutralizing antibodies and their recognized antigenic domains have therefore become the first crucial step for better understanding of the protective antibody response and the rational design of immunogens capable of eliciting the neutralizing antibodies [1–5]. In human immunodeficiency virus type I (HIV-1) infection, viral glycoprotein gp160 that mediates infection of CD4^+^ T lymphocytes is the sole target for neutralizing antibodies. The gp160 is composed of exterior, receptor-binding gp120 and the fusion-mediating, transmembrane gp41 subunits. The unique feature of gp160 is its extensive glycosylation and genetic diversity manifested by rapid generation and high turnover of viral variants during infection [6]. Sequence and structural analysis has revealed the glycosylation and mutations are largely distributed in the hypervarible regions V1–V5 on the exterior surface of gp160 and function to protect the virus from antibody recognition and neutralization [1–5, 7, 8].

Majority of HIV-1 infection is established by one transmitted/founder virus with distinct genetic and phenotypic properties compared to those in the later stages of infection [9–12]. The development of neutralizing antibodies against this virus, however, follows an unusual pathway of inefficiency [2, 4, 13–18]. Most of the antibodies generated during the first few weeks lack neutralizing activities but reactive to gp41 as well as some non-HIV-1 antigens [19–21]. Only after a few months into the infection, autologous neutralizing antibodies become detectable, largely directed to gp120 and invariably strain-specific [4, 13, 14, 22]. Cross-reactive and broadly neutralizing antibodies (bnAbs) capable of neutralizing heterologous viruses across many genetic subtypes can only be generated after years into the infection and most notably in individuals who remain healthy despite prolonged period of infection [1–5, 15, 23]. Isolation and characterization of bnAbs from these individuals have identified five major targets on the gp160. These include the CD4-binding site (CD4bs), the glycan-associated V1V2 and V3/C3 subdomains of gp120, the membrane proximal external regions (MPER) of gp41, and the interface between gp120 and gp41 [1–5, 15]. But how exactly the autologous and bnAbs are generated during the course of HIV-1 infection remain largely unknown. Several elegant studies highlighted the critical role of interplay between viral evolution and antibody development. At the monoclonal levels, germline ancestors for neutralizing antibodies require stimulation by evolving or incoming viral variants during infection [24–29]. Different B cell lineages within the same individuals also appeared to work in concert to drive the development of neutralizing antibodies [25]. At the polyclonal levels, however, dissecting the mechanism underlying the development of neutralizing antibodies is much more complex as polyclonal antibodies function through a dynamic and complex mixture of monoclonal antibodies with diverse targets on the gp160. Studies based on short peptides, chimeric and epitope-specific mutant viruses have identified a few subdomains of gp120 are the major targets for neutralizing activities in polyclonal sera [30–33]. However, the detailed understanding on the scope, specificities and dynamic features of polyclonal antibody recognition against the transmitted/founder virus remain elusive.

Here, we report antibody profiling of sequential plasma samples against transmitted/founder HIV-1 envelope glycoprotein in an epidemiologically linked transmission pair. Using our previously reported approach based on combinatorial antigen library displayed on the surface of the yeast *Saccharomyces cerevisiae*, we were able to delineate polyclonal antibody recognition in both qualitative and quantitative terms [34]. Through sequential analysis of plasma-reactive antigenic sequences over the first 2 years of infection, we decomposed the polyclonal antibody recognition into three major subdomains and showed their development in vivo followed spatiotemporal hierarchy: starting at the ectodomain of gp41, then at the V3C3V4 and V1V2 of gp120. While antibodies to all three subdomains appeared to undergo avidity maturation, the early anti-gp41 antibodies demonstrated no detectable autologous neutralization and only those delayed anti-V3C3V4 and anti-V1V2 antibodies constituted the major neutralizing activities. Our results indicate that the initial antibody response was severely misguided by the transmitted/founder virus and future vaccine design would need to avoid the ectodomain of gp41 and focus on the neutralizing targets in the V3C3V4 and V1V2 subdomains of gp120.

## Results

### Construction and validation of combinatorial antigen library from the transmitted HIV-1 envelopes

**Fig. 1:**
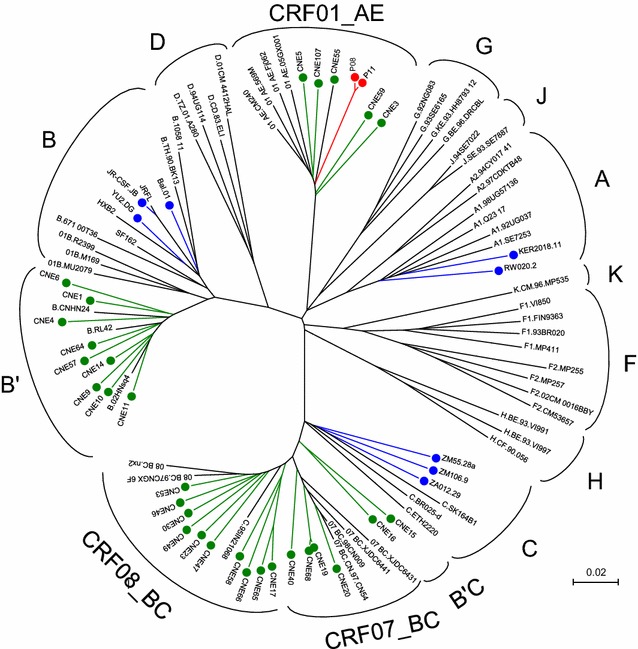
Unrooted neighbor-joining tree depicting the genetic relatedness of full-length envelope sequences from P08 and P11 with the controls. Sequences from P08 and P11 are colored in *red*, those previously obtained from China are in *green*, those provided to us by John Mascola of Vaccine Research Center at NIH are in *blue*, and those commonly used reference sequences for classifying HIV-1 subtypes and CRFs were in *black*. The branch length is drawn to *scale* so that the relatedness between different sequences can be readily assessed. Individual sequences are named at the tip of the branches and their assignment to specific subtype or CRF is also indicated

**Fig. 2:**
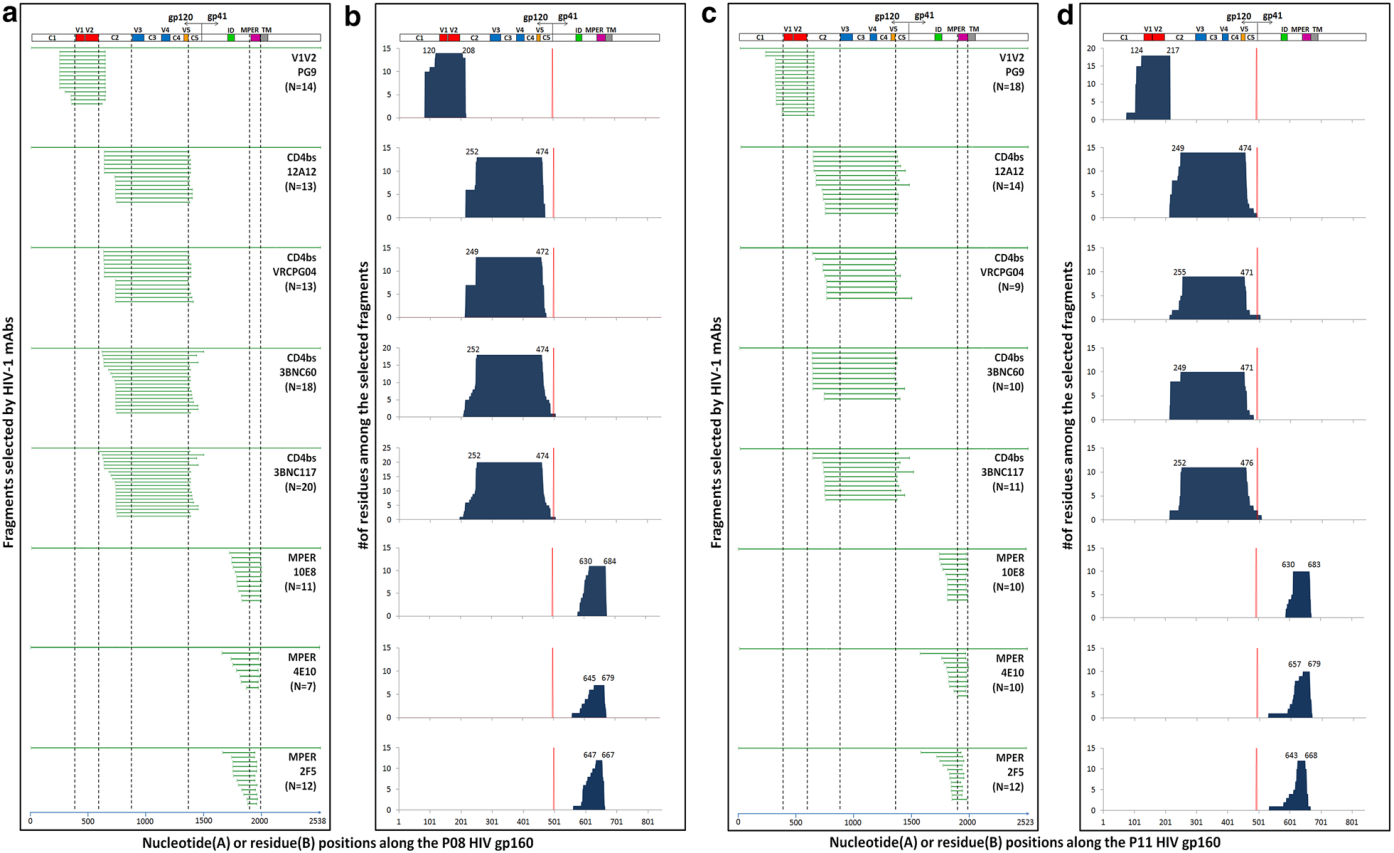
Robustness and specificity of combinatorial library approach in the selection of linear and conformational antigenic fragments by bnAbs with known epitope specificity. **a**, **c** Overlapping sequences of the positive yeast clones selected by bnAbs (PG09, 12A12, VRCPG04, 3BNC60, 3BNC117, 10E8, 4E10, and 2F5) and aligned to the original full-length P08-gp160 and P11-gp160 sequence used in the construction of combinatorial yeast library. *Vertical dashed lines* highlight the V1V2, V3V4V5, and MPER regions in which the epitopes of bnAbs are located. **b**, **d** The number of amino acid residues among the selected fragments along their corresponding positions in the P08 and P11 envelope glycoprotein. The dominant stretch of amino acid residues is numbered on the *top of each graph* according to HXB2 and the *red vertical bars* separate gp120 and gp41. The hypervarible regions V1–V5, interspersed conserved regions C1–C5 in gp120, immune dominant (ID), MPER and transmembrane domain (TM) in gp41 are also indicated

To characterize dynamic changes in antibody recognition during natural infection, we first constructed combinatorial HIV-1 envelope antigen library based on the transmitted/founder viruses from an epidemiologically linked transmission pair P08 and P11. P08 and P11 were identified through a Chinese acute infection cohort for men who have sex with men (MSM) that has been following several thousands of high risk individuals over the last decade. Epidemiologic and clinic documentation indicated that P08 infected P11 during acute infection. The full-length envelope sequences of the transmitted/founder viruses from P08 (P08-gp160) and P11 (P11-gp160) shared 99.9 % homology and phylogenetically grouped with those from the circulating recombinant form 01_AE (CRF01_AE) (Fig. 1). The pseudoviruses bearing the P08-gp160 and P11-gp160 were able to infect the CCR5^+^CD4^+^TZM-bl cell line and demonstrated variable sensitivity to a number of well characterized bnAbs targeted to the vulnerable sites on the envelope (Table 1). The protocol for constructing and validating the combinatorial antigen library has been illustrated in greater details previously [34, 35]. The displayed antigenic fragments were centered around 30–200 residues in length (~100–600 bases) and confirmed to include both linear and conformational epitopes. In this study, eight bnAbs (PG9, 12A12, VRCPG-04, 3BNC60, 3BNC117, 10E8, 4E10, and 2F5) recognizing either conformational or linear epitopes on HIV-1 envelope, were able to select the combinatorial antigen library built with P08-gp160 or P11-gp160 (Fig. 2). Though variable in length, the selected antigenic fragments were clustered together in three distinct regions along the P08-gp160 and P11-gp160 sequences (Fig. 2a, c). The first cluster, selected by PG9, encompassed the V1V2 region of gp120; the second, selected by the CD4bs antibodies 12A12, VRCPG04, 3BNC60, 3BNC117, largely spanned the V3, V4, and V5 regions of gp120; and the third, selected by 10E8, 4E10, and 2F5, covered the MPER region of gp41. The average length of selected antigenic fragments by PG9 was 118 residues, by 12A12, VRCPG04, 3BNC60, 3BNC117 was 234 residues, and by 10E8, 4E10, and 2F5 was 62 residues. Furthermore, the frequency of each amino acid residues among all of the selected fragments was analyzed and plotted along the P08-gp160 and P11-gp160 sequences (Fig. 2b, d). The dominant stretch of residues for each selected antigenic cluster was numerically indicated and invariably covered the epitope specificity previously defined for each of the corresponding bnAbs [36–38]. These results confirmed that the selected antigenic fragments contained both linear and conformational epitopes that were correctly folded and displayed on the surface of the yeast. Furthermore, as PG9 is a glycan-dependent antibody, the selected antigenic fragments must also contain appropriate glycan-moiety for specific recognition. The significant overlap in antigenic fragment sequences with known epitope specificities observed here validated and reinforced the notion that the combinatorial antigenic libraries built with P08-gp160 and P11-gp160 contained both linear and conformational epitopes and were well suited for studying the complex polyclonal antibody recognition during HIV-1 infection.

**Table 1:** Neutralization sensitivity of transmitted/founder P08 and P11 pseudoviruses to various bnAbs

Antibody	Epitope	IC_50_ (µg/ml)	
		P08-gp160	P11-gp160
PG9	V1V2	0.690	1.916
PG16	V1V2	ND	ND
PGT121	Glycan-V3	ND	ND
PGT128	Glycan-V3	0.011	0.015
PGT135	Glycan-V3	ND	ND
2G12	Glycan	ND	ND
b12	CD4bs	ND	ND
VRC01	CD4bs	8.306	6.808
Ibalizumab	CD4	0.027	0.022
2F5	MPER	0.415	2.17
4E10	MPER	0.571	0.568
10E8	MPER	0.131	0.217

*ND* not detectable

### Spatiotemporal hierarchy in antibody development against distinct subdomains of HIV-1 envelope during natural infection

**Fig. 3:**
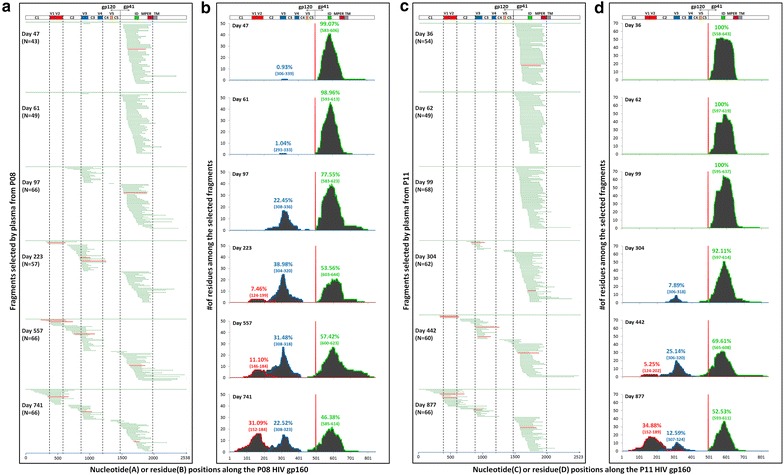
Analysis of major antigenic subdomains on the transmitted/founder HIV-1 envelope based on the positive yeast clones selected by the sequential plasma samples from P08 and P11. **a**, **c** Overlapping sequences of the positive yeast clones selected by the sequential plasma samples and aligned to the original full-length P08-gp160 and P11-gp160 sequence used in the construction of combinatorial yeast library. *Vertical dashed lines* highlight the V1V2, V3C3V4, and the ectodomain of gp41 in which the selected fragments are predominantly located. The *four red horizontal lines* from each subdomain represent the selected clones for studying avidity maturation of plasma antibodies during infection (see Fig. 5). **b**, **d** Quantitative measurement of antibody recognition against distinct subdomains on transmitted/founder HIV-1 envelope during natural infection. Each subdomain was identified based on the frequency of amino acid residues among the selected fragments and colored coded. The locations of the most frequently recognized residues within V1V2, V3C3V4 and ectodomain of gp41 are indicated in *parentheses* according to HXB2 and highlighted in *red*, *blue*, and *green*, respectively. The percentages represent the area under the curve (AUC) of each antigenic subdomain verse the total AUC (*shaded area*). The hypervarible regions V1–V5, interspersed conserved regions C1–C5 in gp120, immune dominant (ID), MPER and transmembrane domain (TM) in gp41 are also indicated

Next, we used six sequential plasma samples from P08 and P11 over 2 years after infection to select the antigenic fragments from the combinatorial libraries of transmitted/founder P08-gp160 and P11-gp160, respectively. A total of 706 overlapping fragments were selected by the sequential plasma samples and their distribution over different time points and along the P08-gp160 and P11-gp160 sequences are shown in Fig. 3a, c. Clearly, there was an intrinsic hierarchy in the emergence of antibody recognition against distinct envelope subdomains during infection. In P08, for instance, almost all fragments selected by plasma on day 47 and 61 after infection were confined to the ectodomain of gp41. By day 97, however, fragments largely containing V3, C3 and V4 (V3C3V4) in gp120 became detectable accompanied by substantial decline in proportion of gp41 fragments. By day 223, while increase in V3C3V4 and decrease in gp41 fragments continued, the V1V2 fragments began to appear. The proportion of the V1V2 fragment continued to increase by day 557 and 741 while those V3C3V4 and gp41 fragments decreased (Fig. 3a). Similar hierarchy in antibody emergence and recognition was also found in P11 despite the dominant recognition against gp41 fragments was prolonged and that against V3C3V4 and V1V2 were substantially delayed compared to that of P08 (Fig. 3c). Of note, the size of selected fragment varied from 13 to 265 residues but mostly centered between 30 and 200 residues, consistent with our initial design of the library for containing both linear and conformational epitopes [34, 35].

**Fig. 4:**
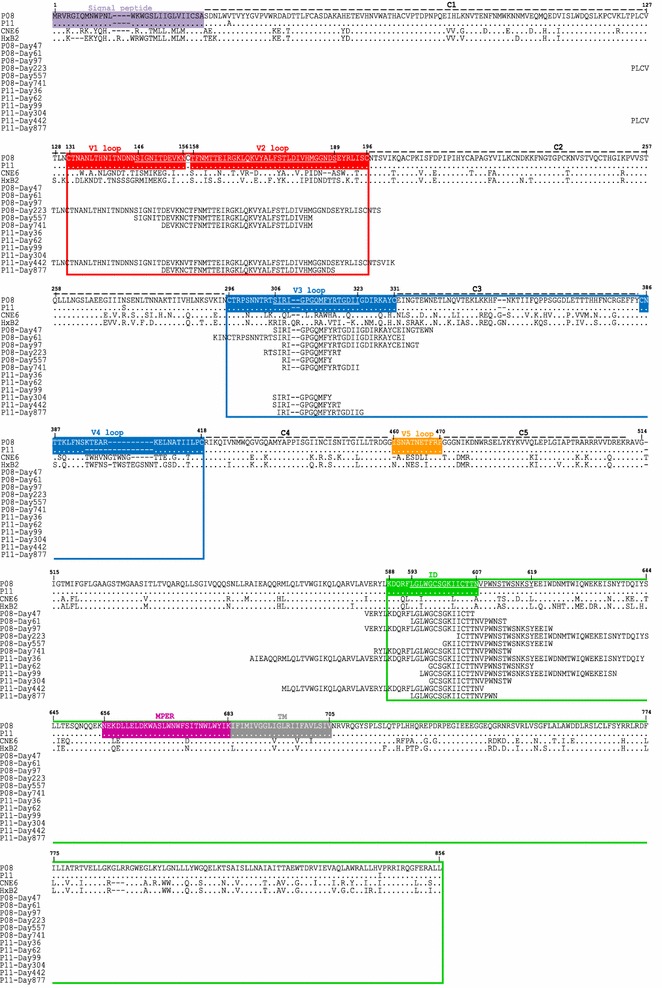
The most frequently recognized amino acid sequences on the envelope of P08 and P11. The sequences are aligned to the full-length transmitted/founder HIV-1 envelope from P08 and P11 and compared with and numbered according to HXB2. The predominantly recognized amino acid sequences are *underlines*. Those highlighted in *red*, *blue* and *green boxes* represent the piece of sequences transplanted into the CNE6 backbone to generate various chimeric viruses. The signal peptide, hypervarible regions V1–V5, interspersed conserved regions C1–C5 in gp120, immune dominant (ID), MPER and transmembrane domain (TM) in gp41 are also indicated

To characterize the antibody recognition in more quantitative manner, the frequency of each amino acid residues among all of the selected fragments was analyzed and plotted along the P08-gp160 and P11-gp160 sequences (Fig. 3b, d). Similar to the dynamic changes in proportion and location of selected fragments, the total number of amino acids recognized by the plasma, as reflected by the area under the curve (AUC), also underwent rapid turnover. In P08, for example, about 99 % antibody recognition was directed to the ectodomain of gp41 on day 47 and 61 after infection (Fig. 3b). Sequence analysis revealed a stretch of amino acid residues LGLWGCSGKIICTTNVPWNSTWSNKSY (aa593–619) (Fig. 4) was the most frequently recognized which in fact corresponded to the dominant epitope previously identified and included in the diagnostic kit for HIV-1 infection [39]. As disease progressed to day 97, antibody recognition shifted substantially to gp120 with over 22 % directed to V3C3V4 and concomitant decline to 78 % against the ectodomain of gp41. Sequence analysis identified the most frequently recognized residues in the V3C3V4 were SIRIGPGQMFYRTGDII (aa306–323) (Fig. 4), consistent with earlier findings where the tip of the V3 loop was the most immune dominant in gp120 [40]. By day 223, a sizable fraction of antibody recognition began to be expanded to V1V2 (7 %) while that to V3C3V4 increased to 39 % and to gp41 declined to 54 %. V1V2 recognition continued to increase to 11 % by day 557 and 31 % by day 741 after infection while that against V3C3V4 and gp41 demonstrated appreciable trend of decline (Fig. 3b). The most frequently recognized residues in V1V2 were SIGNITDEVKNCTF**N**MTTEIRGKLQKVYALFSTLDIVHMGGNDS (aa146–189) (Fig. 4) which included the epitope recognized by glycan-dependent bnAb PG09 and those isolated from RV144 Thai HIV-1 vaccinees and naturally infected individuals [24, 41]. Similar pattern of antibody recognition was also recapitulated in P11 despite the emergence of antibody recognition against V3C3V4 and V1V2 was substantially delayed compared to that in P08 (Fig. 3d). These results clearly indicate a unique spatiotemporal hierarchy in antibody development against distinct subdomains of transmitted/founder HIV-1 envelope during natural infection.

### Avidity maturation of antibody recognition against distinct subdomains of HIV-1 envelope during natural infection

**Fig. 5:**
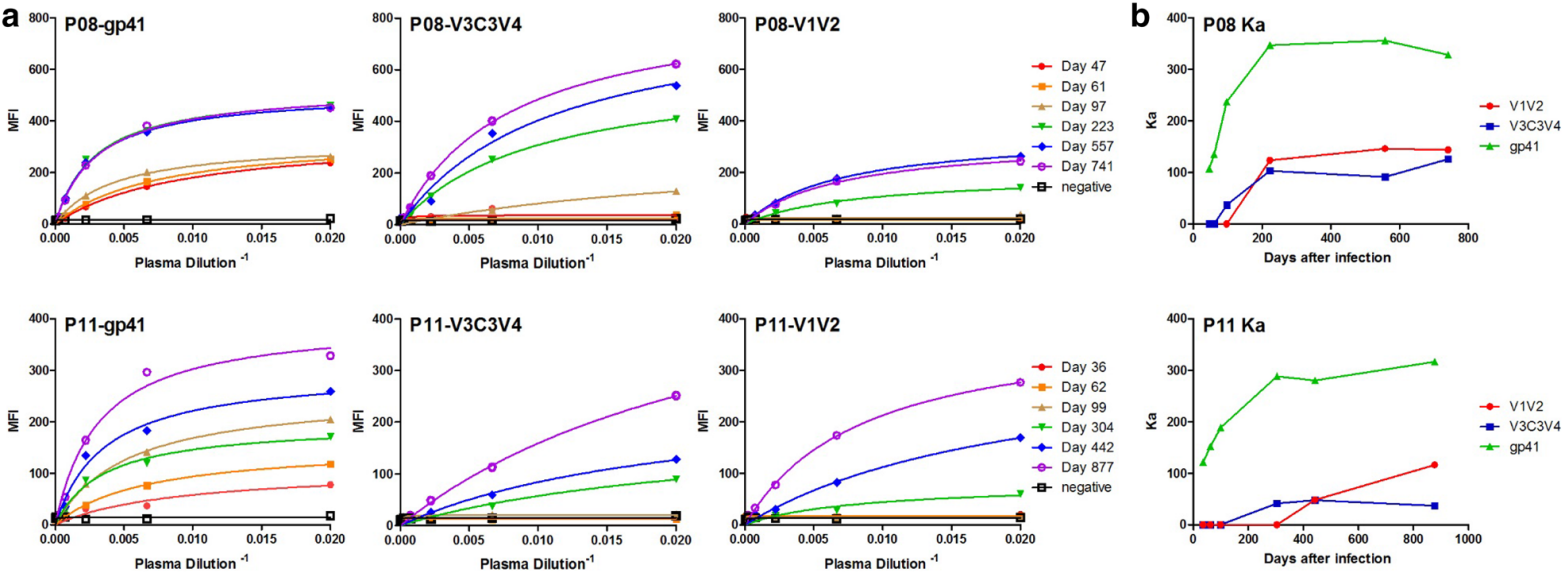
Avidity maturation of subdomain-specific antibodies during natural HIV-1 infection. **a** For each distinct subdomain, four representative yeast clones were used (see Fig. 3a, c) to measure the mean fluorescence intensity (MFI) of all sequential plasma samples from P08 and P11, and from which the association (Ka) and disassociation (Kd) constants were calculated using nonlinear least squares fit. **b** Dynamic changes in Ka of subdomain-specific antibodies during the first 2–2.4 years of HIV-1 infection. Ka is the reciprocal of Kd

As antibody recognition against the ectodomain of gp41, V3C3V4 and V1V2 appeared in an evolving fashion over the course of infection, we went further to ask whether such recognition was associated with avidity maturation in the corresponding plasma. The term of “avidity maturation” is used here to define the composite binding activity of polyclonal antibodies in the plasma versus “affinity maturation” of a monoclonal antibody commonly used in the field. To this end, we measured the mean fluorescence intensity (MFI) and calculated association constant (Ka) against the ectodomain of gp41, V3C3V4 and V1V2 for the sequential plasma samples from both P08 and P11. Specifically, we selected representative yeast clones expressing the distinct envelope subdomain (highlighted in red in Fig. 3a, c) and mixed with a serial threefold dilution of the sequential plasma samples from P08 and P11. After 1 h incubation at room temperature and thorough washes with cold phosphate-buffered saline (PBS), the antibody recognition of yeast clones expressing the distinct cluster of envelope fragments were detected by FACS. The MFI was recorded, plotted and fitted using a nonlinear least square curve against the reciprocal plasma dilution (Fig. 5a). The association constant (Ka) was then calculated for each plasma samples and plotted over the study period (Fig. 5b). In both P08 and P11, a general trend of increase in MFI was found against the ectodomain of gp41, V3C3V4 and V1V2 despite of their differences in the temporal emergence during the course of infection (Fig. 5a). During the first 3 months of infection, MFI against gp41 displayed significantly higher levels than that against V3C3V4 and V1V2. It was not until the later points when MFI against V3C3V4 and V1V2 became detectable and substantially increased. In cases such as P08-V1V2 and P08-gp41, the MFI appeared to be saturated during later stage of infection, reflected by the overlapping curves for the last two or three samples. Furthermore, the calculated Ka also demonstrated a trend of increase over the course of infection (Fig. 5b). In particular, the rise in Ka against the ectodomain of gp41 was more profound during the first year after infection than the ensuing period when the Ka became relatively stable. Similar trend was also identified for Ka against V3C3V4 and V1V2 although the number of measurable Ka was rather limited (Fig. 5b). Taken together, these results suggested that while the antibody development against the ectodomain of gp41, V3C3V4 and V1V2 followed a spatiotemporal order, there was concurrent avidity maturation against all three subdomains in the corresponding plasma. Such maturation process appeared to undertake a nonlinear process with relative faster early phase followed by a relative stable one. Similar trend of maturation process was recently reported for VRC01, a bnAb against CD4bs, during natural infection [28].

**Fig. 6:**
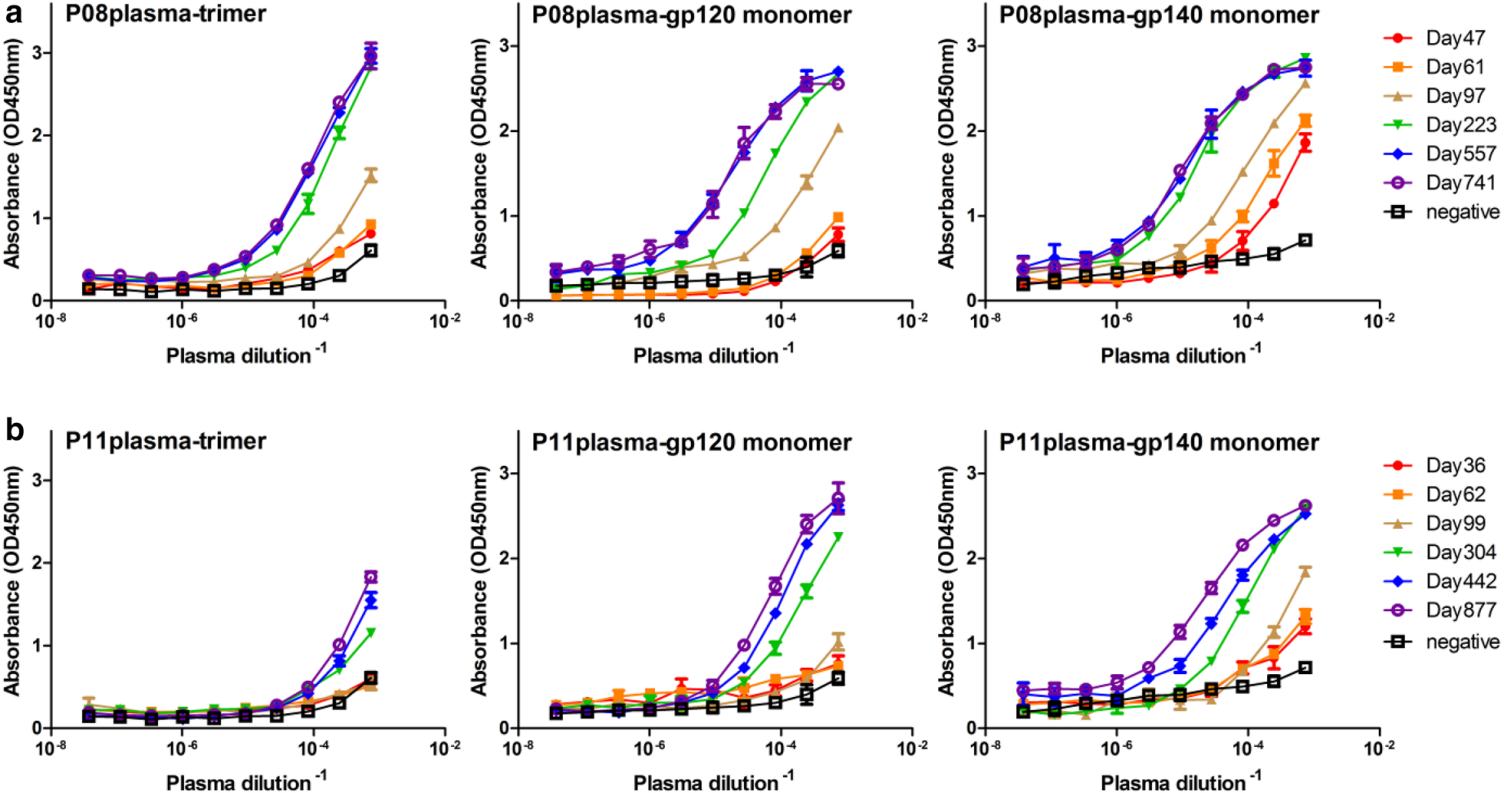
Standard ELISA assay to measure the dynamic changes in plasma antibody binding of P08 (**a**) and P11 (**b**) against three forms of envelope glycoprotein; trimeric ectodomain of NL4-3 (subtype B) constructed based on BG505 SOSIP.664, monomeric gp120 and monomeric gp140 (CRF01_AE)

We also used standard ELISA assay to measure the dynamic changes in antibody binding against three forms of heterologous envelope glycoprotein; trimeric ectodomain of NL4-3 (subtype B) constructed based on BG505 SOSIP.664 [42], monomeric gp120 and monomeric gp140 (CRF01_AE). As shown in Fig. 6, there is a clear trend of increase in antibody binding against all forms of envelope glycoprotein over the course of infection. The last two or three plasma samples, in particular from P08, demonstrated saturation effect reflected by their overlapping binding curves (Fig. 6a), consistent with relatively stabilized binding avidity observed above against the three distinct subdomains on the envelope glycoprotein.

### V3C3V4 and V1V2 but not gp41 contain the major targets for autologous neutralization

**Fig. 7:**
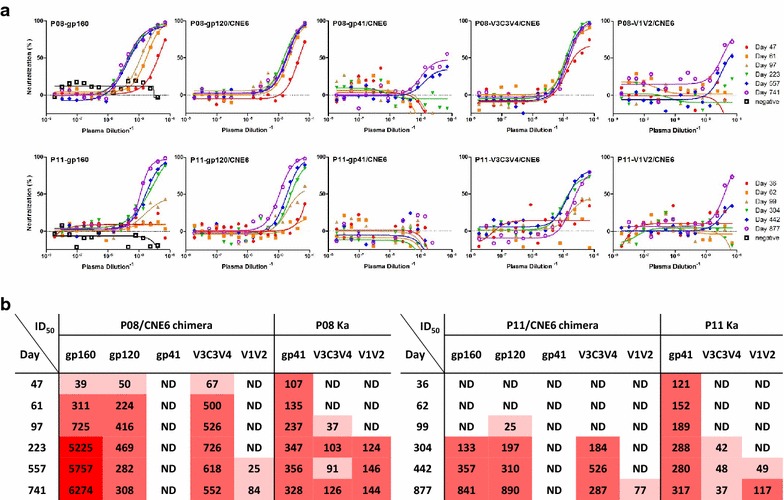
Determination of major antigenic subdomains for autologous neutralization by comparing neutralizing sensitivity of parental and subdomain-specific chimeras to sequential plasma samples from P08 and P11. **a** Percent neutralization on the *y* axis indicates the reduction in luciferase activity in the presence of plasma verse those without. **b** Half-maximal inhibitory dilutions (ID_50_) were calculated as the dilution at which the plasma demonstrated inhibition of 50 % infection compared with the controls. The actual Ka values for corresponding plasma samples against each subdomain were also included for comparative purpose. *Different color* intensities correspond to different ID_50_ and Ka values for clarity, *lighter* for lower and *darker* for higher. *ND* not detectable

While binding antibody displayed spatiotemporal hierarchy and avidity maturation during infection, their contribution to overall neutralization remained unclear. To address this question, we generated a series of chimeric viruses for gp41, V3C3V4, V1V2 and gp120 derived from P08-gp160 and P11-gp160. We chose one of our previously characterized full-length envelopes (CNE6) as the backbone as it was able to confer complete resistance to P08 and P11 plasma neutralization (Additional file 1: Figure S1) [43]. We reasoned that if any of the chimeric viruses became susceptible to P08 or P11 plasma neutralization, the replaced region should contain the targets for autologous neutralization. To this end, we tested neutralization sensitivity of all chimeric viruses, along with the parental ones, against autologous sequential plasma samples from P08 and P11. As shown in Fig. 7a, b, replacement of gp120 but not gp41 in the CNE6 backbone substantially transferred the neutralization sensitivity to P08 and P11 plasma, suggesting a major proportion of autologous neutralization was mediated by anti-gp120 antibodies. In P08, autologous neutralization against gp120-chimeras was found to increase until the day 223 and then declined in the ensuing period, perhaps due to the emergence and turnover of viral variants (Fig. 7b). Within gp120, a large fraction of autologous neutralization appeared to be contributed by anti-V3C3V4 antibodies and that by anti-V1V2 antibodies only occurred at much later stage of infection. For example, when the V3C3V4 region from P08-gp160 or P11-gp160 was inserted into the CNE6 backbone, the chimeras acquired neutralization sensitivity to P08 and P11 plasma to the levels that were comparable to those inserted with entire gp120 (Fig. 7b). Furthermore, acquisition of neutralization sensitivity by the V1V2 chimera was much delayed till day 557 in P08 and day 877 in P11 after the initial infection (Fig. 7b). Overall, the rise in neutralization sensitivity of V3C3V4 and V1V2 chimeras appeared to temporally coincide with increase in Ka values against the same regions measured by MFI (Fig. 7b), suggesting increases in neutralizing activity is likely due to increases in binding avidity against neutralizing epitopes within V3C3V4 and V1V2. Collectively, these data indicated that anti-gp120 and in particular anti-V3C3V4 and V1V2 antibodies were the major source of autologous neutralization. In contrast, anti-gp41 antibodies, despite of its early emergence and dominance during infection, contributed bare minimum to the overall autologous neutralization.

## Discussion

We report here the systematic characterization of antibody recognition against transmitted/founder HIV-1 envelope glycoprotein during natural infection in an epidemiologically linked transmission pair infected by highly homologous CRF01_AE strains. Based on several complementary approaches to determine the specificities of binding as well as neutralizing antibodies, we were able to decompose the complex plasma antibody recognition into three discrete subdomains on the HIV-1 envelope: ectodomain of gp41, V3C3V4 and V1V2 of gp120. The development of these subdomain-specific antibodies appeared to follow a spatiotemporal hierarchy with distinct dynamic, biochemical and neutralizing properties. While antibodies to all three subdomains appeared to undergo avidity maturation, the early and strong anti-gp41 antibodies failed to translate into detectable autologous neutralization. Instead, it was the much delayed anti-V3C3V4 and anti-V1V2 antibodies constituted the major neutralizing activities. In particular, it reinforced the early discoveries in that the majority of the initial antibody response was severely misguided by the transmitted/founder virus towards its gp41 subdomain and therefore missed the most critical window of opportunity to contain or clear the virus replication through recognizing the neutralizing epitopes in the V3C3V4 and V1V2 subdomains [19, 20]. By the time when the neutralizing antibody response was indeed mounted in a substantial manner, it was much too late and virus had already established its permanent residence in the target cells. Such defects in mistargeting and mistiming have provided some explanations for the failure of human immune system to contain viral replication during early infection, and strongly recommend that future vaccine design would need to avoid the ectodomain of gp41 and focus more on those neutralizing targets in the V3C3V4 and V1V2 subdomains of gp120.

At the current stage, we are uncertain about the underlying mechanisms leading to the spatiotemporal hierarchy for antibody recognition against the three major envelope subdomains. The overwhelming response against gp41 during early infection could be due to the pre-existing gp41 cross-reactive memory B cells that acquired reactivity with autologous gp41 [19, 44, 45]. A recent study showing majority of gut-derived anti-gp41 antibodies cross-reacted with commensal bacteria supports this hypothesis [21]. It could also be due to the shedding of gp120 leading to the exposure of preferred structures during early infection although the exact step and timing of such preference during viral replication are currently unknown. Generally speaking, gp41 exhibits at least three distinct conformational states during the viral fusion process: the prefusion, the prehairpin intermediate, and the postfusion conformation. It is believed that the conformational differences among the three states are so great that each of them likely presents distinct antigenic surface to the immune system [46–48]. So far, only the prehairpin intermediate was found to be the target of bnAbs such as 2F5, 4E10 and 10E8 while the other two states were largely recognized by non-neutralizing antibodies. In particular, the non-neutralizing antibodies against gp41 appeared to group in two clusters based on the location of their respective epitopes. Cluster I antibodies recognize the immunodominant C–C loop of gp41 (aa590–600), and the cluster II antibodies react with the downstream immunodominant segment (aa644–663) [46–49]. But whether the two clusters of antibodies specifically react with prefusion and postfusion conformation remain to be determined. As the antibody recognition found in our study subjects overlapped with cluster I antibodies, the conformational state against which they were initially generated was unlikely to be the prehairpin intermediate. Whatever the conformational state was recognized, it must be the one to be avoided in our vaccine design to prevent non-neutralizing epitopes as well as severe misguidance and mistiming found during natural infection.

Consistent with earlier reports, the appearance of autologous neutralizing antibodies occurred not until 2–3 months into the infection [4, 14, 17, 22]. The major proportion was clearly contributed by the anti-V3C3V4, rather than by the anti-V1V2 antibodies. Such contribution appeared to be persistent throughout the infection but with the trend of increase during the first year and then decrease in the ensuing period of infection, perhaps due to the emergence and turnover of viral variants. Furthermore, anti-V3C3V4 neutralizing antibody response was also strain-specific as plasma samples from both P08 and P11 failed to demonstrate cross neutralizing activities against a panel of heterologous viruses (Table 2). These results indicate that the variable rather than the conserved structures within the V3C3V4 region were the major targets of autologous neutralization. Clearly, they were unlikely to include the type of epitopes recognized by the CD4bs bnAbs as the fragments selected by the plasma samples only encompassed V3C3V4 and devoid of V5 region (Fig. 2). Through sequence analysis of full-length envelope genes from the sequential plasma samples, we found that the V3 loop remained relative conserved up to the time point when decline in autologous neutralizing activity was found (Additional file 1: Figure S2). The downstream C3 and V4 regions, however, displayed considerate degree of variation including both point mutations as well as length polymorphism (Additional file 1: Figure S2). Whether the C3 or V4 region alone or in combination were the major neutralization target and drove the viral escape from the autologous neutralization requires further investigation, although a prior study of subtype C infected individuals suggested the case [22, 50]. In addition, we were puzzled by the belated emergence of anti-V1V2 neutralizing antibody compared to those in subtype B and C infected individuals [22, 24, 51–53]. It could be related to the viral subtype CRF01_AE or specific structural features of the envelope studied in our study subjects. Similar studies involving more subjects infected with different viral subtypes will help to address this important question. At the least, it was unlikely the result of structural abnormality (Additional file 1: Figure S3) or major defects in infectivity of chimeric viruses (Additional file 1: Figure S4). Of note, the most frequently recognized stretch of residues in the V1V2 by the autologous plasma samples overlapped with the epitopes recognized by glycan-dependent bnAbs and those isolated from RV144 Thai HIV-1 vaccinees and naturally infected individuals [24, 41, 53]. It is uncertain whether such overlapping nature in epitopes would trigger further maturation of strain-specific antibodies to become bnAb during the later stage of infection. The intricate and delicate interplay between evolving viral variants and developing B cell lineages would ultimately determine such likelihood [24–26]. But nevertheless, as V1V2 is the major target for both strain-specific as well as bnAbs, it should certainly serve as one of the vulnerable targets for future vaccine design. Lastly, it needs to be pointed out that from the technical point of view, the combinational library-based approach offers unprecedented advantages in probing the antibody recognition with qualitative and quantitative specificities. However, it is by no means perfect. It is expected that some of the conformational epitopes, in particular those formed through inter-molecular interaction, would be lost during the fragmentation process for library construction. Some subdominant epitopes, although may be important for neutralizing antibodies, could also be rendered undetectable. We have to bear these caveats in mind when we analyze and interpret the data.

**Table 2:** Neutralizing activity of P08 and P11 plasma samples against a panel of pseudoviruses with distinct genotypic and phenotypic features from China and abroad

Strain	Clade	Origin	ID_50_	
			P08-plasma	P11-plasma
P08-gp160	CRF01_AE	China	5000	3645
P11-gp160	CRF01_AE	China	2500	1193
CNE5	CRF01_AE	China	ND	ND
CNE55	CRF01_AE	China	45	ND
CNE59	CRF01_AE	China	463	ND
CNE6	B′	China	ND	ND
CNE11	B′	China	ND	ND
CNE14	B′	China	156	ND
CNE15	B′C	China	ND	ND
CNE20	CRF07_BC	China	ND	ND
CNE30	CRF08_BC	China	ND	ND
HxB2	B	France	135	ND
JRFL	B	USA	ND	ND
SF162	B	USA	45	ND
KER2018.11	A	Kenya	ND	ND
RW020.2	A	Rwanda	ND	ND
Bal.01	B	USA	ND	ND
YU2.DG	B	USA	ND	ND
JR-CSF.JB	B	USA	ND	ND
ZA012.29	C	South Africa	ND	ND
ZM106.9	C	Zambia	ND	ND
ZM55.28a	C	Zambia	ND	ND

*ND* not detectable

## Conclusion

Our study has unraveled the complex and dynamic feature of antibody development against transmitted/founder HIV-1 envelope glycoprotein during natural infection. The major binding and neutralizing antigenic subdomains identified here will provide critical reference for our better understanding of the spatiotemporal feature of protective antibody response during natural infection and assist our rational design of vaccines that will empower the strengths while minimize the weaknesses of human immune recognition.

## Methods

### Study subjects and plasma samples

Two acutely infected individuals, P08 and P11, were chosen for the study. P08 was 37 and P11 38 years old when identified through China’s largest acute infection cohort for man who have sex with man (MSM) that followed several thousands of high risk individuals over the last decade. P08 and P11 were epidemiologically linked transmission pair and P08 infected P11 during acute infection based on epidemiologic and clinic documentation. When enrolled on day 30 after infection for P08 and day 18 for P11, both individuals were negative for HIV-1 antibody measured by enzyme-linked immunosorbent assay (ELISA) and indeterminate Western blot test, and therefore fell into Fiebig II-IV substage of acute infection [54]. P08 and P11 had baseline CD4 lymphocyte count of 339 and 369 per cubic millimeter (FACS Calibur, BD) and plasma viral load of 30,600 and 889,000 RNA copies per milliliter (Cobas AmpliPrep/Cobas TaqMan HIV-1 version 5.1 Assay, Roche), respectively. Both individuals progressed to diseases relatively fast and by 2 years into the infection, the CD4 lymphocyte count dropped to 147 for P08 and 181 for P11 per cubic millimeter and plasma viral load remained as high as 12,500 for P08 and 40,429 for P11 RNA copies per milliliter. Sequential plasma and peripheral blood mononuclear cells (PBMCs) were collected over the first 2 years of infection and stored at −80 °C until use. Neither P08 nor P11 received any antiretroviral therapy during the study period. This study was reviewed and approved by the institutional research ethics committee at the No. 1 Hospital of China Medical University in Shenyang, Liaoning Province, China.

### Full-length envelopes, phylogenetic analysis, pseudoviruses and neutralization assay

The full-length envelope genes from P08 (P08-gp160) and P11 (P11-gp160) were obtained through PCR amplification of single HIV-1 RNA molecules directly from the plasma samples. The reference envelopes from subtype A (KER2018.11 and RW020.2), subtype B (JRFL, Bal.01, YU2.DG) and subtype C (ZA012.29, ZM106.9, ZM55.28a) were kindly provided by John Mascola of Vaccine Research Center at National Institute of Health (NIH). The representative envelopes from HIV-1 infected individuals in China came from our previously studies including those from CRF01_AE, subtype B′, subtype B′C, and CRF07_BC and CRF_08BC. These full-length envelope sequences were aligned using the Clustal W program together with selected subtypes/CRFs of geographical importance from the Genbank database. Phylogenetic analysis was conducted using the neighbor-joining method and the reliability of the branching orders was tested by bootstrap analysis of 1000 replicates [55].

These envelope clones were also used to generate pseudoviruses by co-transfection with backbone construct pNL43R-E-luciferase into the 293 cells. Forty-eight hours later, the culture supernatant containing the pseudoviruses was collected and tested for luciferase activity to standardize viral input in the subsequent neutralization analysis. Neutralizing activities of plasma samples from P08 and P11 and neutralizing sensitivity of P08 and P11 pseudoviruses to various bnAbs were analyzed as previously described [43]. In brief, 100 TCID_50_ of pseudoviruses was incubated with serially diluted plasma, or various bnAb in a 96-well plate in triplicate for 1 h at 37 °C. Approximately 2 × 10^4^ TZM-bl cells were then added and the cultures were maintained for an additional 48 h at 37 °C. Neutralizing activity was measured by the reduction in luciferase activity compared with controls (Bright-Glo luciferase assay system, E2650, Promega). Half-maximal inhibitory concentrations or dilutions (IC_50_ or ID_50_) were reported as the concentration for bnAbs or dilution for plasma required to inhibit infection by 50 % compared with the controls. The values were calculated using the dose–response inhibition model with a variable slope in GraphPad Prism, version 5.0 (GraphPad Software Inc., La Jolla, CA, USA). The bnAb PG9 and PG16 were kindly provided by Wayne Koff at International AIDS Vaccine Initiative (IAVI), VRC01 by John Mascola of Vaccine Research Center at NIH, 3BNC117 by Michel Nussenzweig at Rockefeller University, and Ibalizumab by David Ho at Aaron Diamond AIDS Research Center of Rockefeller University. The rest of the bnAbs were obtained from NIH reference and reagents program.

### Generation and verification of envelope chimeras

Chimeric gp160 envelopes were generated using an overlapping PCR strategy with gp120, gp41, V3C3V4, V1V2 subdomains amplified separately and then together with the flanking regions from the backbone of CNE6. The resultant envelope chimeras were cloned into the pcDNA3.1 (Invitrogen), verified by sequencing before used for pseudovirus production. Sensitivity of chimeric pseudoviruses to plasma neutralization was measured as described above.

### Construction and expression of transmitted/founder HIV-1 envelope combinatorial libraries on the surface of yeast *Saccharomyces cerevisiae*

Construction of yeast library displaying the combinatorial antigens was carried out as described previously [34]. In brief, the full-length P08 and P11 envelope gene from the day 30 and day 18 after infection, respectively, was amplified by single molecule PCR, purified (QIAquick DNA purification kit, QIAGEN) and digested by DNase I into fragments about 50 base pair (bp) in length. The digested fragments were reassembled to approximately 100–600 bp fragments through controlled number of PCR cycles, added A-tails (DNA A-tailing kit, TAKARA) and ligated to the modified yeast surface displayed vector pCTCON2-T. The ligation products were transformed into the *Escherichia coli* competent cells, amplified, extracted and then further transformed into the competent yeast cell line EBY100 using electroporation. Transformed yeast cells were partially spread on SDCAA Amp plates and incubated overnight at 30 °C to estimate the number and insert sequences of colonies for quality control purpose. Conditions for yeast growing and induction of surface antigen expression in solution have been previously described [34]. In short, EBY100 yeasts were first grown in SDCAA media at 30 °C for 48 h. At the exponential growth phase, yeasts were transferred to SGCAA media for induction of antigen expression at 20 °C for 48 h before incubating with either plasma samples or monoclonal antibodies for subsequent analysis [34].

### Immunofluorescence staining, sorting, sequencing and sequence analysis of bnAb- or plasma-reactive yeast clones by FACS

The entire procedure was conducted as previously described [34]. Induced yeast cells (10^6^–10^7^) were collected by centrifugation (6000 rmp/s, 1 min), washed twice with cold PBS and incubated with either bnAb or patient plasma (1:100 dilution) on ice for 1 h with occasional agitation. After washed three times with cold PBS, the cells were incubated with PE labeled anti-human IgG secondary antibody (1:200 dilution, rabbit anti-human IgG-PE, Santa Cruz) on ice for another 45 min, washed again with PBS for three times, analyzed and sorted for positive clones using FACS Aria II (BD, USA). The positive yeast clones were grown in SDCAA before plasmids were extracted (Yeast plasmid kit, Omega Bio-Tek) for sequencing and sequence analysis (Sequencher 5.0, Gene Codes Corp.). The most frequently recognized amino acid residues within each subdomain were calculated as over 90 % percentile among the selected fragments for each subdomain.

### Measurement of Kd and Ka for each antigenic subdomain

The technique relies on measuring the MFI of the bound polyclonal antibodies, at and variety of concentrations of polyclonal antibodies, on the c-myc positive yeast [56]. Specifically, four representative yeast clones were selected from each envelope subdomain based on their coverage and mixed in equal proportion (10^6^ cells) before incubated with a serial 1:3 dilution of sequential plasma samples from P08 and P11. After washed twice with cold PBS, the mixture was resuspended in PE labeled anti-human secondary antibody (1:200 dilution, rabbit anti-human IgG-PE, Santa Cruz) and incubated for another hour. Antibody recognition of yeast clones expressing the distinct envelope subdomains were detected by FACS. The MFI was recorded, plotted and fitted using a nonlinear least square curve against the reciprocal plasma dilution. Kd value was determined using the following equation: y = MFImax × Plasmadilution^−1^/(Kd + Plasmadilution^−1^). Ka is the reciprocal of Kd.

### Measurement of plasma binding to trimeric and monomeric gp120 and gp140 by ELISA

Trimeric ectodomain of NL4-3 (subtype B) constructed based on BG505 SOSIP.664 [42] was kindly provided by Dr. Yi Shi at Institute of Microbiology, Chinese Academy of Sciences. Monomeric gp120 and monomeric gp140 (CRF01_AE) derived from a CRF01_AE circulating strain CM235 (Genebank #: AAG28611) isolated in Thailand in year 2000 were purchased and produced from 293T cells (Immune Technology Corp, China). Recombinant envelope glycoprotein was coated overnight at 4 °C on the 96-well plate (100 ng/well), blocked for 2 h at 37 °C with 1 % bovine serum albumin (BSA) in PBS before addition of ten serial threefold dilutions of plasma samples. After incubating for 1 h at 37 °C and three-time thorough washes with PBST (PBS with 0.05 % Tween), the secondary antibody conjugated with horseradish peroxidase (1:4000 dilution, anti-human IgG-HRP, Promega) was added before applying substrate for detectioin. Maximum absorbance at 450 nm and corresponding plasma dilution were recorded (Microplate Reader, Bio-Rad). Plasma samples from HIV-1 negative individuals were included as negative controls.

## Additional file

10.1186/s12977-016-0243-2 Figure S1 Neutralization resistance of pseudovirus bearing the CNE6 Env to the polyclonal plasma samples from P08 (A) and P11 (B). Figure S2 Alignment of a total of 137 full-length envelope sequences from the sequential plasma samples of P08 (A) and P11 (B) over the course of natural infection. Dates of sampling are indicated on the left. Dots represent identical residues, while dashes represent gaps introduced to preserve the alignment.  Asterisks indicate the premature stops. Signal peptide, hypervariable (V1, V2, V3, V4, and V5) and conserved (C1, C2, C3, C4, C5) regions, Loop D, CD4-binding loop in gp120, fusion peptide (FP), amino- and carboxy-terminal heptad repeat (NHR and CHR) sequences, immunodominant region (ID), membrane-proximal external region (MPER) and transmembrane domain (TM) in gp41 are highlighted along the sequences. Figure S3 Standard ELISA assay to measure the binding of sequential plasma samples from P08 and P11 (A, C) and selected mAbs (B, D) to soluble gp120 captured from the original (P08 and P11) and chimeric viruses (P08/CNE6 and P11/CNE6). Figure S4 Infectivity of chimeric viruses (P08/CNE6 and P11/CNE6) compared with the parental ones from P08 (A) and P11 (B) as well as control CNE6.
