# Supplementary material for: Spatiotemporal hierarchy in antibody recognition against transmitted HIV-1 envelope glycoprotein during natural infection
Source: Retrovirology. 2016 Feb 17;13:12. doi: 10.1186/s12977-016-0243-3 (PMC4756523; doi:10.1186/s12977-016-0243-3)
Supplement: Supplementary file 1 — 10.1186/s12977-016-0243-2 Figure S1 Neutralization resistance of pseudovirus bearing the CNE6 Env to the polyclonal plasma samples from P08 (A) and P11 (B). Figure S2 Alignment of a total of 137 full-length envelope sequences from the sequential plasma samples of P08 (A) and P11 (B) over the course of natural infection. Dates of sampling are indicated on the left. Dots represent identical residues, while dashes represent gaps introduced to preserve the alignment. Asterisks indicate the premature stops. Signal peptide, hypervariable (V1, V2, V3, V4, and V5) and conserved (C1, C2, C3, C4, C5) regions, Loop D, CD4-binding loop in gp120, fusion peptide (FP), amino- and carboxy-terminal heptad repeat (NHR and CHR) sequences, immunodominant region (ID), membrane-proximal external region (MPER) and transmembrane domain (TM) in gp41 are highlighted along the sequences. Figure S3 Standard ELISA assay to measure the binding of sequential plasma samples from P08 and P11 (A, C) and selected mAbs (B, D) to soluble gp120 captured from the original (P08 and P11) and chimeric viruses (P08/CNE6 and P11/CNE6). Figure S4 Infectivity of chimeric viruses (P08/CNE6 and P11/CNE6) compared with the parental ones from P08 (A) and P11 (B) as well as control CNE6. [file 12977_2016_243_MOESM1_ESM.pdf]

Figure S1 Neutralization resistance of pseudovirus bearing the CNE6 Env to the polyclonal plasma samples from P08 (A) and P11 (B).

Figure S2 Alignment of a total of 137 full-length envelope sequences from the sequential plasma samples of P08 (A) and P11 (B) over the course of natural infection. Dates of sampling are indicated on the left. Dots represent identical residues, while dashes represent gaps introduced to preserve the alignment. Asterisks indicate the premature stops. Signal peptide, hypervariable (V1, V2, V3, V4, and V5) and conserved (C1, C2, C3, C4, C5) regions, Loop D, CD4-binding loop in gp120, fusion peptide (FP), amino- and carboxy-terminal heptad repeat (NHR and CHR) sequences, immunodominant region (ID), membrane-proximal external region (MPER) and transmembrane domain (TM) in gp41 are highlighted along the sequences.

Figure S3 Standard ELISA assay to measure the binding of sequential plasma samples from P08 and P11 (A, C) and selected mAbs (B, D) to soluble gp120 captured from the original (P08 and P11) and chimeric viruses (P08/CNE6 and P11/CNE6).

Figure S4 Infectivity of chimeric viruses (P08/CNE6 and P11/CNE6) compared with the parental ones from P08 (A) and P11 (B) as well as control CNE6.

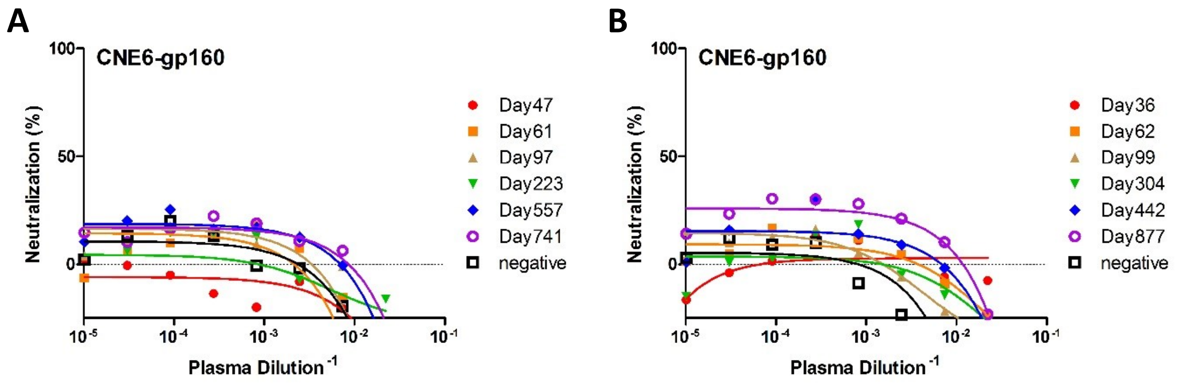
A
CNE6-gp160
Neutralization (%)
Plasma Dilution -1
Day47
Day61
Day97
Day223
Day557
Day741
negative
B
CNE6-gp160
Neutralization (%)
Plasma Dilution -1
Day36
Day62
Day99
Day304
Day442
Day877
negative

Figure S1

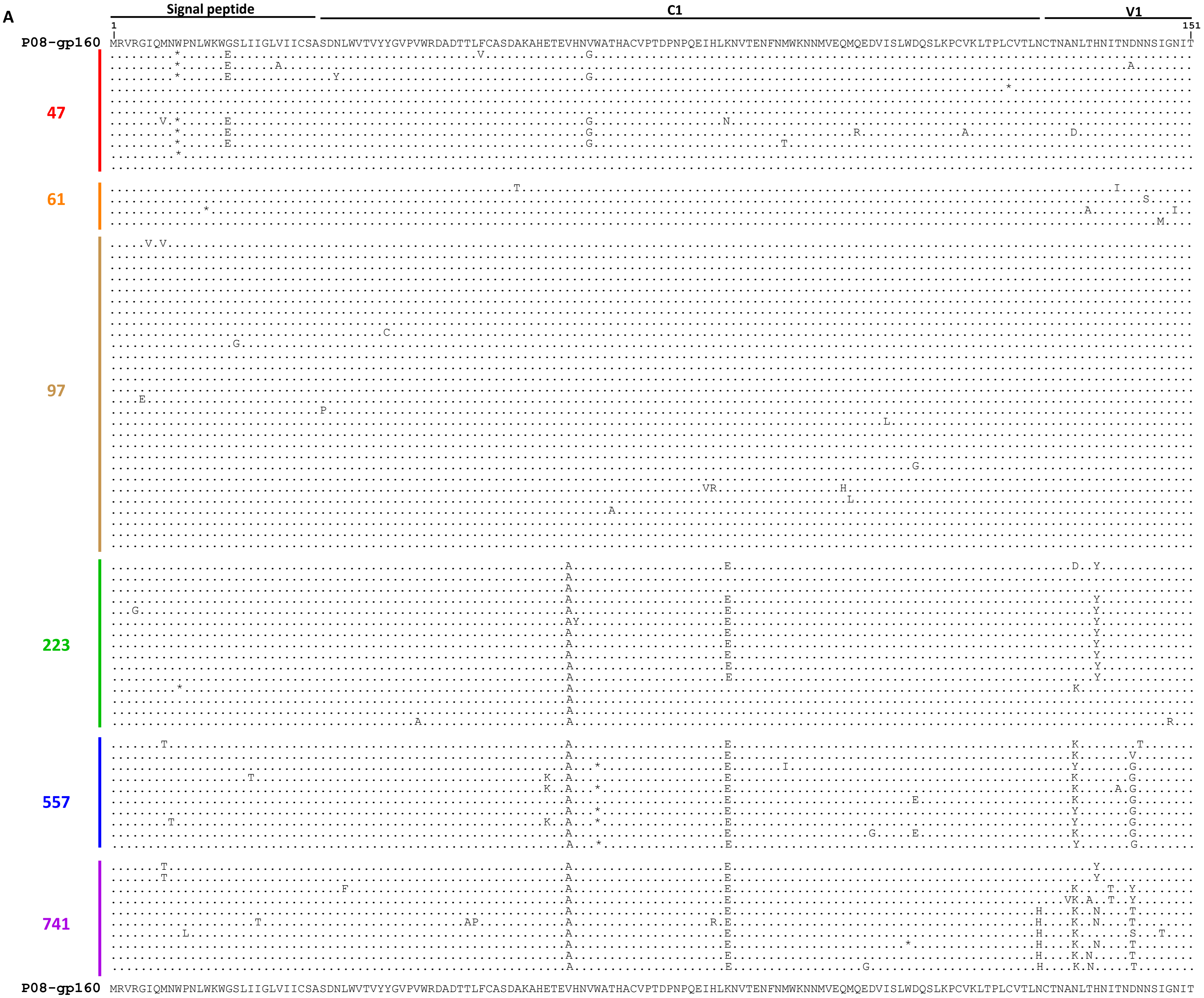
A
Signal peptide
C1
V1
1
151
P08-gp160 MRVRGIQMNWPNLWKWGSLIIGLVIICSASDNLWVTVYYGVPVWRDADTTLFCASDAKAHETEVHNVWATHACVPTDPNPQEIHLKNVTENFNMWKNNMVEQMQEDVISLWDQSLKPCVKLTPLCVTLNCTNANLTHNITNDNNSIGNIT
47
61
97
223
557
741
P08-gp160 MRVRGIQMNWPNLWKWGSLIIGLVIICSASDNLWVTVYYGVPVWRDADTTLFCASDAKAHETEVHNVWATHACVPTDPNPQEIHLKNVTENFNMWKNNMVEQMQEDVISLWDQSLKPCVKLTPLCVTLNCTNANLTHNITNDNNSIGNIT

Figure S2

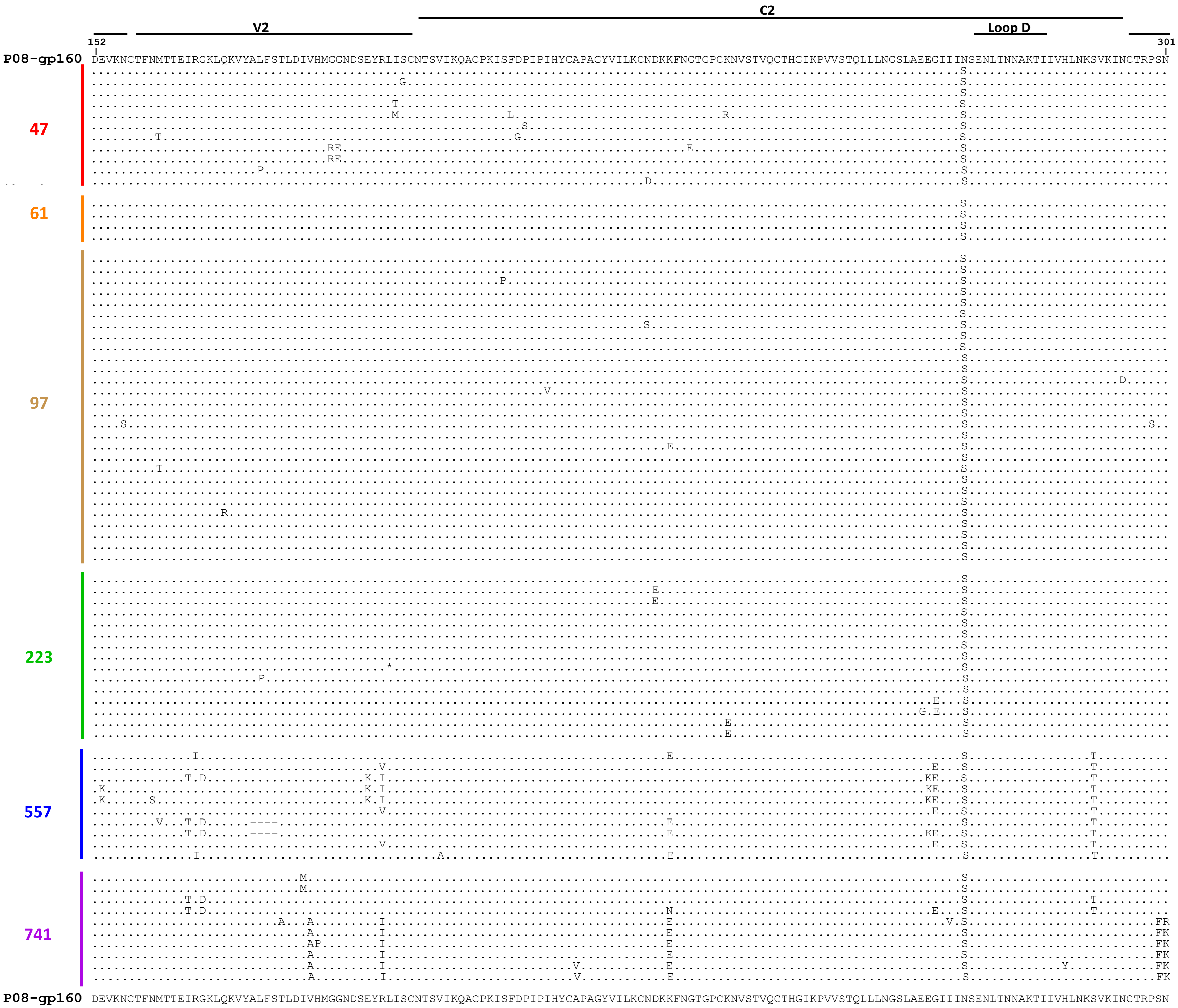
C2
V2
Loop D
152
301
P08-gp160 DEVKNCTFNMTTEIRGKLQKVYALFSTLDIVHMGGNDSEYRLISCNTSVIKQACPKISFDPIPIHYCAPAGYVILKCNDKKFNGTGPCKNVSTVQCTHGIKPVVSTQLLLNGSLAEEGIIINSENLTNNAKTIIVHLNKSVKINCTRPSN
47
61
97
223
557
741
P08-gp160 DEVKNCTFNMTTEIRGKLQKVYALFSTLDIVHMGGNDSEYRLISCNTSVIKQACPKISFDPIPIHYCAPAGYVILKCNDKKFNGTGPCKNVSTVQCTHGIKPVVSTQLLLNGSLAEEGIIINSENLTNNAKTIIVHLNKSVKINCTRPSN

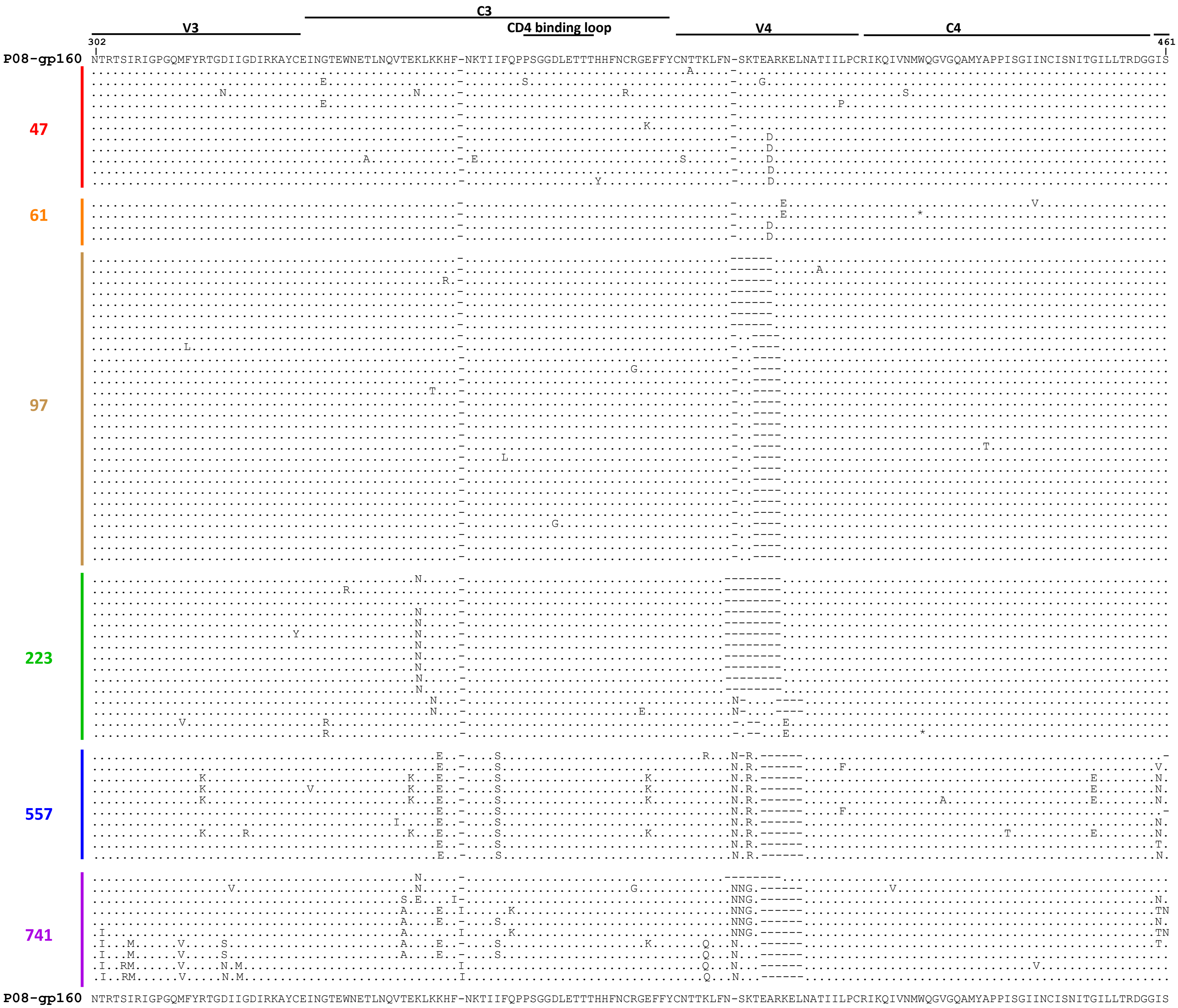
C3
V3
CD4 binding loop
V4
C4
302
461
P08-gp160 NTRTSIRIGPGQMFYRTGDIIGDIRKAYCEINGTEWNETLNQVTEKLKKHF-NKTIIFQPPSGGDLETTTHHFNCRGEFFYCNTTKLFN-SKTEARKELNATIILPCRIKQIVNMWQGVGQAMYAPPISGIINCISNITGILLTRDGGIS
47
61
97
223
557
741
P08-gp160 NTRTSIRIGPGQMFYRTGDIIGDIRKAYCEINGTEWNETLNQVTEKLKKHF-NKTIIFQPPSGGDLETTTHHFNCRGEFFYCNTTKLFN-SKTEARKELNATIILPCRIKQIVNMWQGVGQAMYAPPISGIINCISNITGILLTRDGGIS

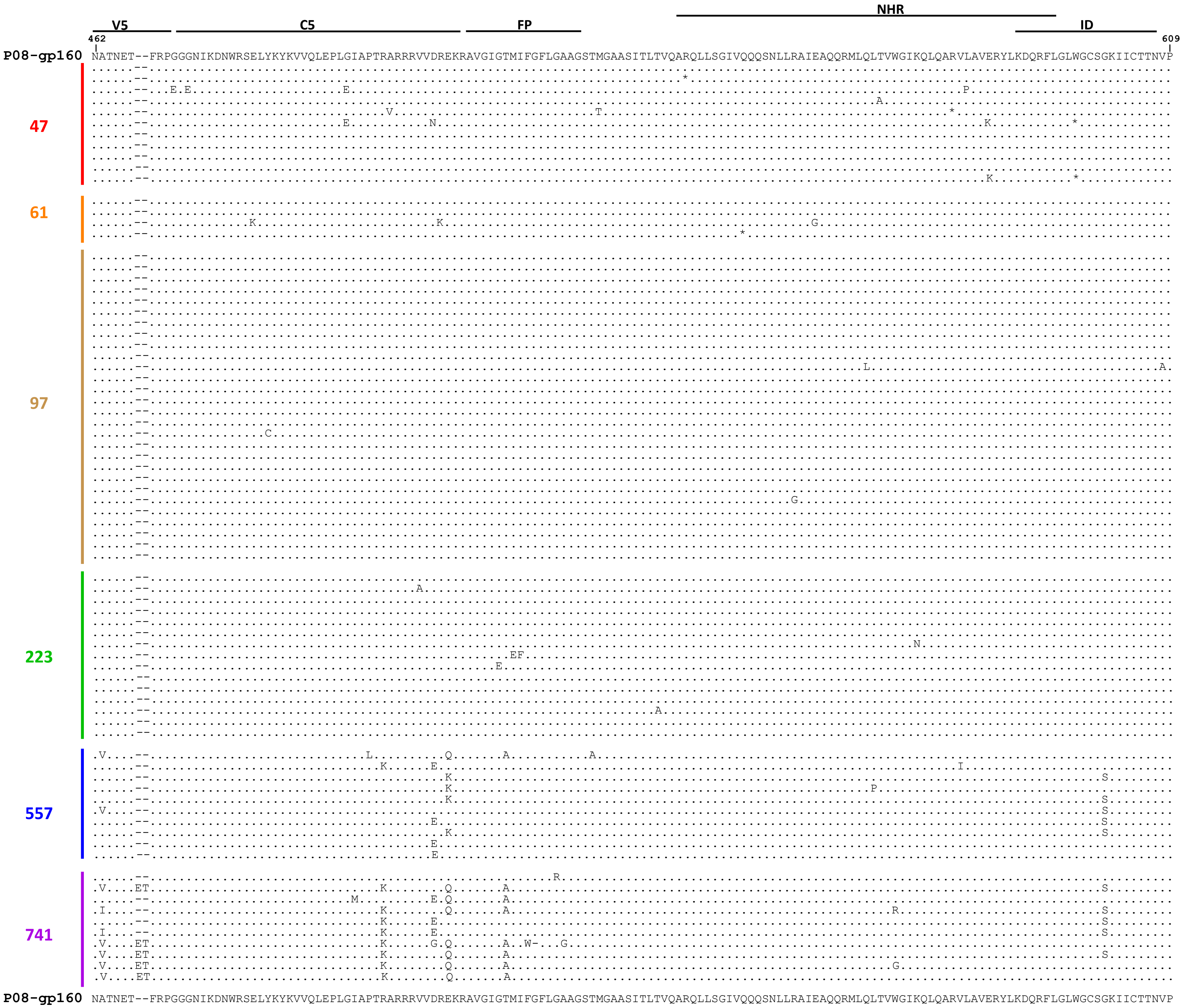
V5
C5
FP
NHR
ID
462
609
P08-gp160 NATNET--FRPGGGNIKDNWRSELYKYKVVQLEPLGIAPTRARRRVVDREKRAVGIGTMIFGFLGAAGSTMGAASITLTVQARQLLSGIVQQQSNLLRAIEAQQRMLQLTVWGIKQLQARVLAVERYLKDQRFLGLWGCSGKIICTTNVP
47
61
97
223
557
741
P08-gp160 NATNET--FRPGGGNIKDNWRSELYKYKVVQLEPLGIAPTRARRRVVDREKRAVGIGTMIFGFLGAAGSTMGAASITLTVQARQLLSGIVQQQSNLLRAIEAQQRMLQLTVWGIKQLQARVLAVERYLKDQRFLGLWGCSGKIICTTNVP

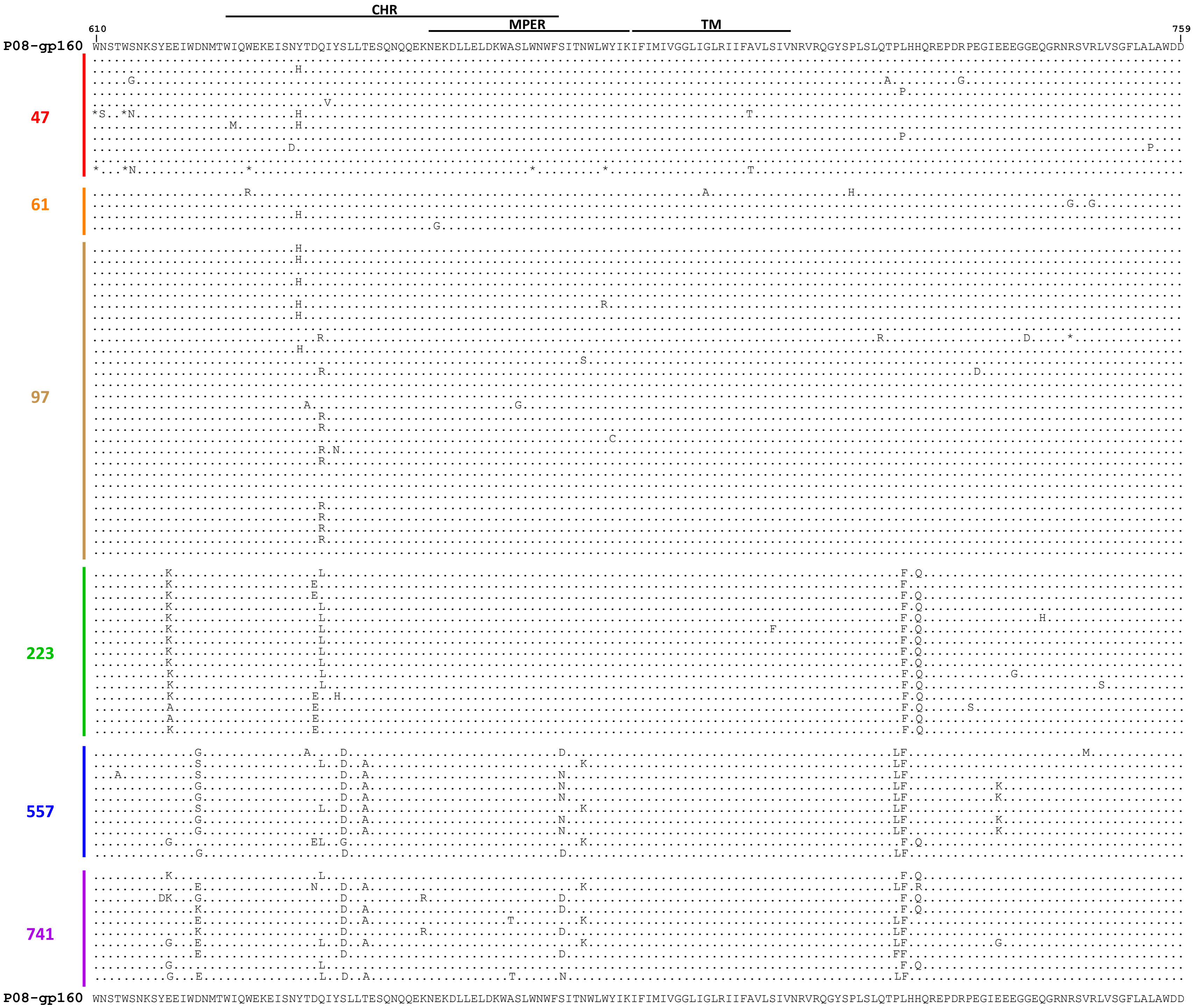
CHR
MPER
TM
610
759
P08-gp160 WNSTWSNKSYEEIWDNMTWIQWEKEISNYTDQIYSLLTESQNQQEKNEKDLLELDKWASLWNWFSITNWLWYIKIFIMIVGGLIGLRIIFAVLSIVNRVRQGYSPLSLQTPLHHQREPDRPEGIEEEGGEQGRNRSVRLVSGFLALAWDD
47
61
97
223
557
741
P08-gp160 WNSTWSNKSYEEIWDNMTWIQWEKEISNYTDQIYSLLTESQNQQEKNEKDLLELDKWASLWNWFSITNWLWYIKIFIMIVGGLIGLRIIFAVLSIVNRVRQGYSPLSLQTPLHHQREPDRPEGIEEEGGEQGRNRSVRLVSGFLALAWDD

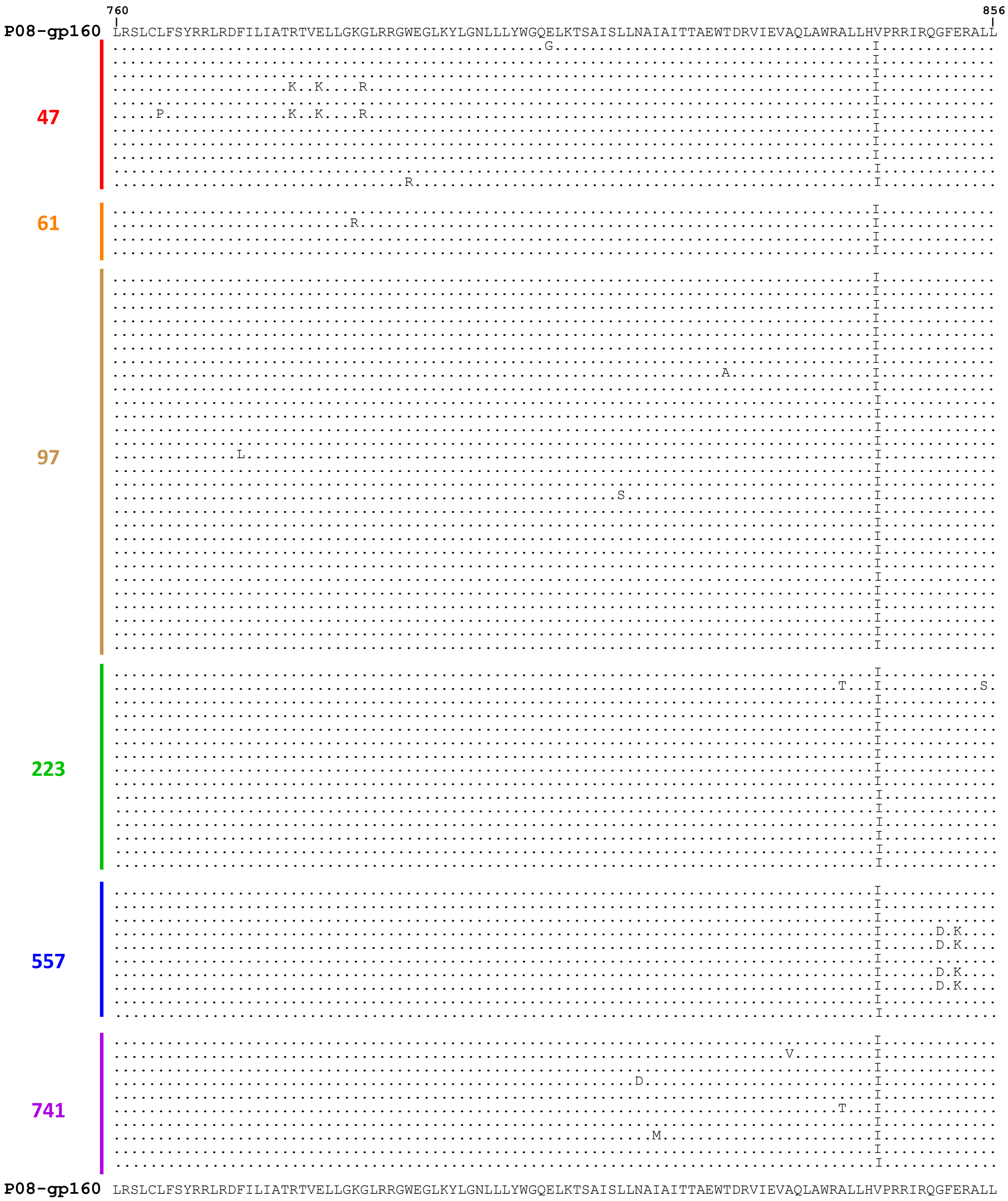
760
856
P08-gp160 LRSLCLFSYRRLRDFILIATRTVELLGKGLRRGWEGLKYLGNLLLYWGQELKTSAISLLNAIAITTAEWTDRVIEVAQLAWRALLHVPRRIRQGFERALL
47
61
97
223
557
741
P08-gp160 LRSLCLFSYRRLRDFILIATRTVELLGKGLRRGWEGLKYLGNLLLYWGQELKTSAISLLNAIAITTAEWTDRVIEVAQLAWRALLHVPRRIRQGFERALL

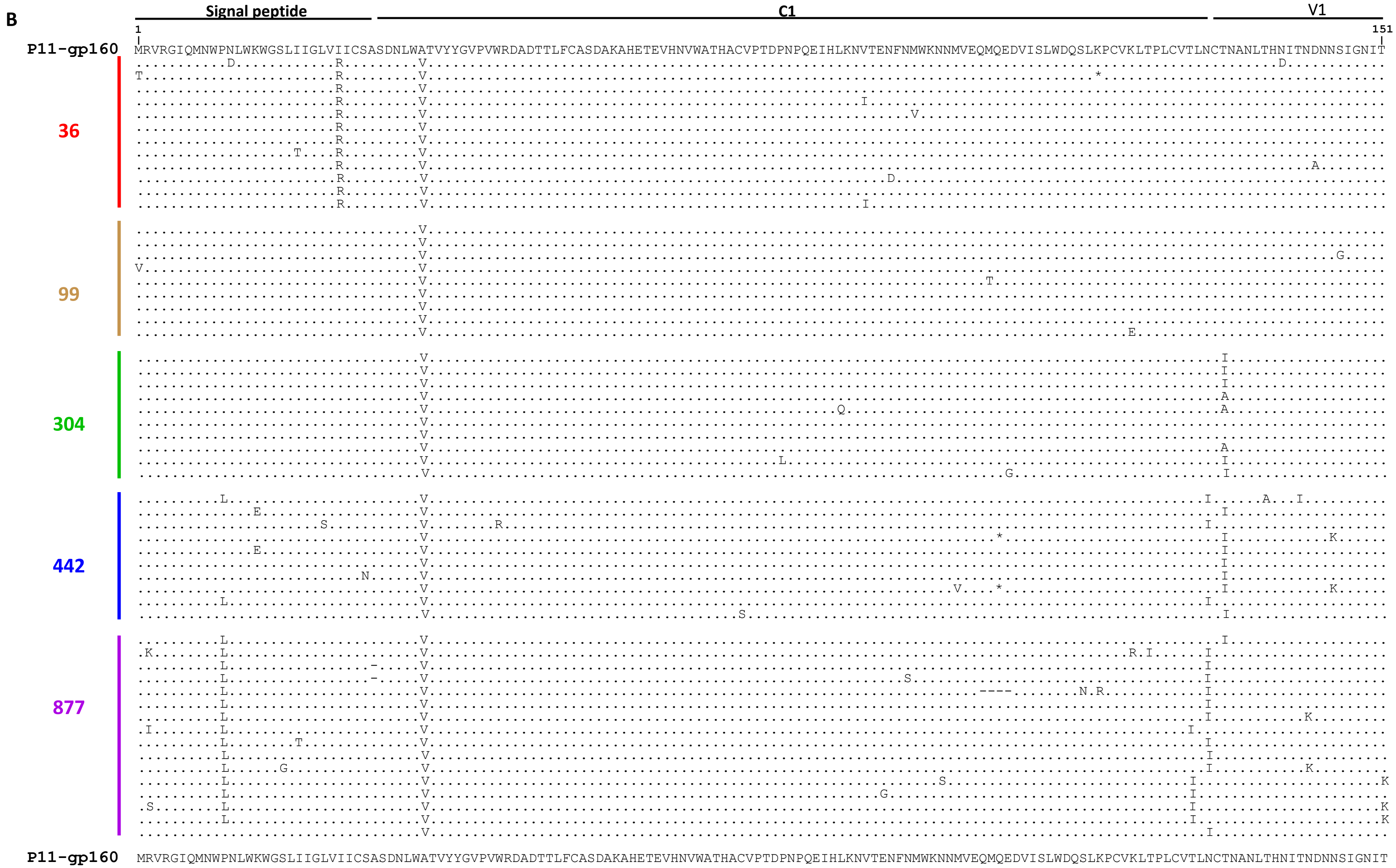
B
Signal peptide
C1
V1
1
151
P11-gp160
MRVRGIQMNWPNLWKWGSLIIGLVIICSASDNLWATVYYGVPVWRDADTTLFCASDAKAHETEVHNVWATHACVPTDPNPQEIHLKNVTENFNMWKNNMVEQMQEDVISLWDQSLKPCVKLTPLCVTLNCTNANLTHNITNDNNSIGNIT
36
99
304
442
877
P11-gp160
MRVRGIQMNWPNLWKWGSLIIGLVIICSASDNLWATVYYGVPVWRDADTTLFCASDAKAHETEVHNVWATHACVPTDPNPQEIHLKNVTENFNMWKNNMVEQMQEDVISLWDQSLKPCVKLTPLCVTLNCTNANLTHNITNDNNSIGNIT

Figure S2

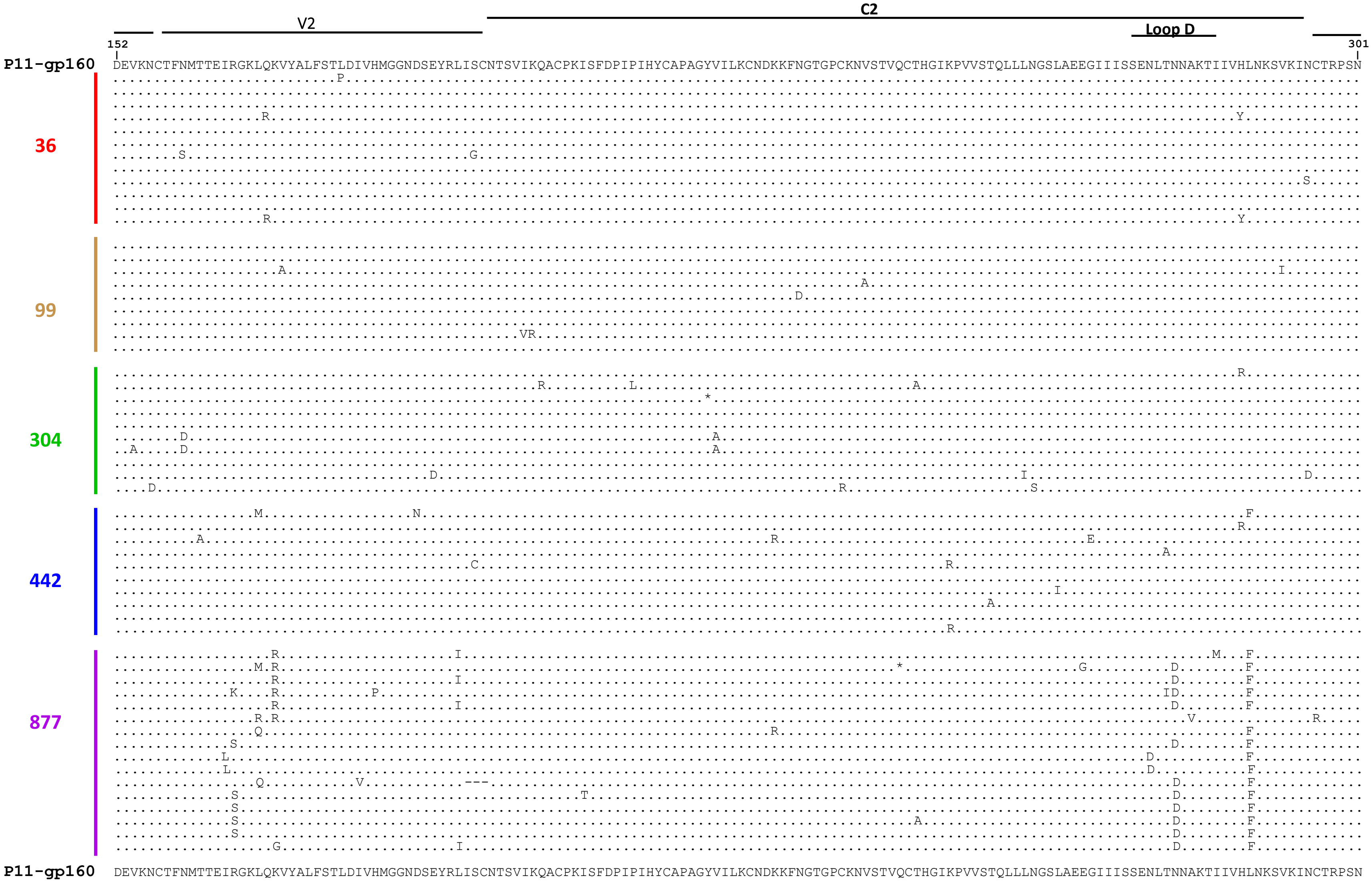

C2
V2
Loop D
152
301
P11-gp160
DEVKNCTFNMTTEIRGKLQKVYALFSTLDIVHMGGNDSEYRLISCNTSVIKQACPKISFDPIPIHYCAPAGYVILKCNDKKFNGTGPCKNVSTVQCTHGIKPVVSTQLLLNGSLAEEGIIISSENLTNNAKTIIVHLNKSVKINCTRPSN
36
99
304
442
877
P11-gp160
DEVKNCTFNMTTEIRGKLQKVYALFSTLDIVHMGGNDSEYRLISCNTSVIKQACPKISFDPIPIHYCAPAGYVILKCNDKKFNGTGPCKNVSTVQCTHGIKPVVSTQLLLNGSLAEEGIIISSENLTNNAKTIIVHLNKSVKINCTRPSN

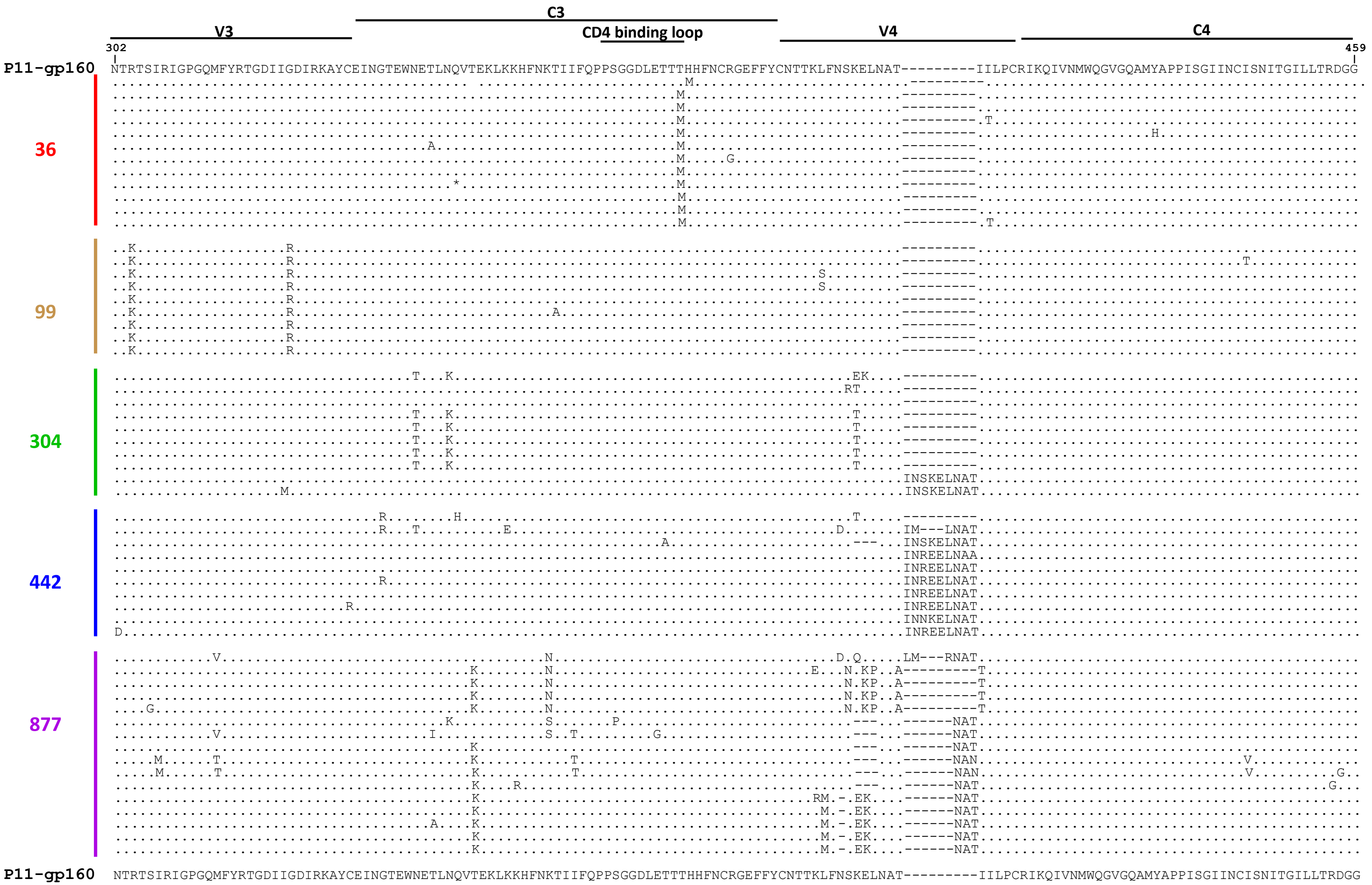

C3
V3
CD4 binding loop
V4
C4
302
459
P11-gp160
NTRTSIRIGPGQMFYRTGDIIGDIRKAYCEINGTEWNETLNQVTEKLKKHFNKTIIFQPPSGGDLETTTHHFNCRGEFFYCNTTKLFNSKELNAT---------IILPCRIKQIVNMWQGVGQAMYAPPISGIINCISNITGILLTRDGG
36
99
304
442
877
P11-gp160
NTRTSIRIGPGQMFYRTGDIIGDIRKAYCEINGTEWNETLNQVTEKLKKHFNKTIIFQPPSGGDLETTTHHFNCRGEFFYCNTTKLFNSKELNAT---------IILPCRIKQIVNMWQGVGQAMYAPPISGIINCISNITGILLTRDGG

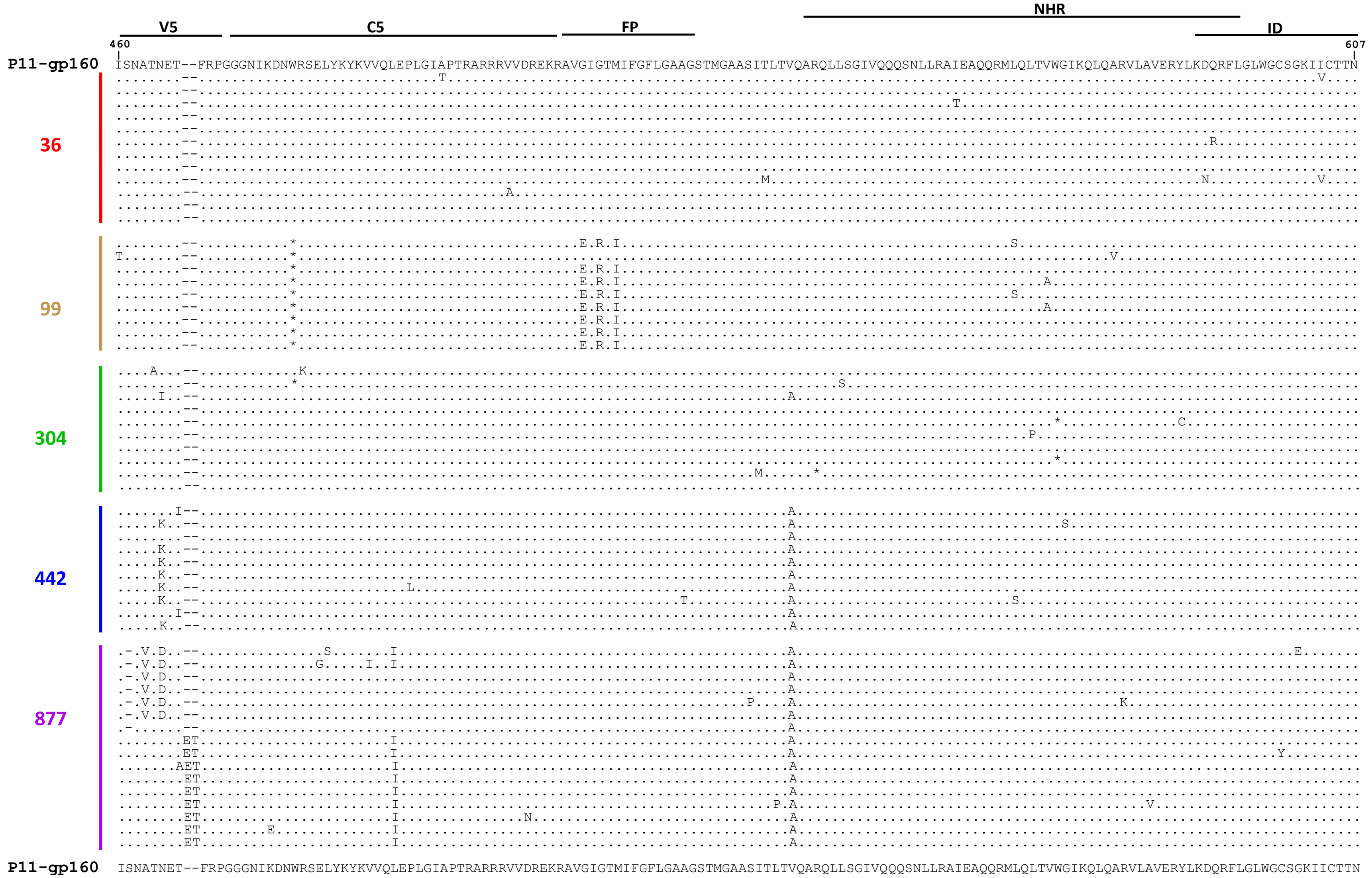
NHR
V5
C5
FP
ID
460
607
P11-gp160 ISNATNET--FRPGGGNIKDNWRSELYKYKVVQLEPLGIAPTRARRRVVDREKRAVGIGTMIFGFLGAAGSTMGAASITLTVQARQLLSGIVQQQSNLLRAIEAQQRMLQLTVWGIKQLQARVLAVERYLKDQRFLGLWGCSGKIICTTN
36
99
304
442
877
P11-gp160 ISNATNET--FRPGGGNIKDNWRSELYKYKVVQLEPLGIAPTRARRRVVDREKRAVGIGTMIFGFLGAAGSTMGAASITLTVQARQLLSGIVQQQSNLLRAIEAQQRMLQLTVWGIKQLQARVLAVERYLKDQRFLGLWGCSGKIICTTN

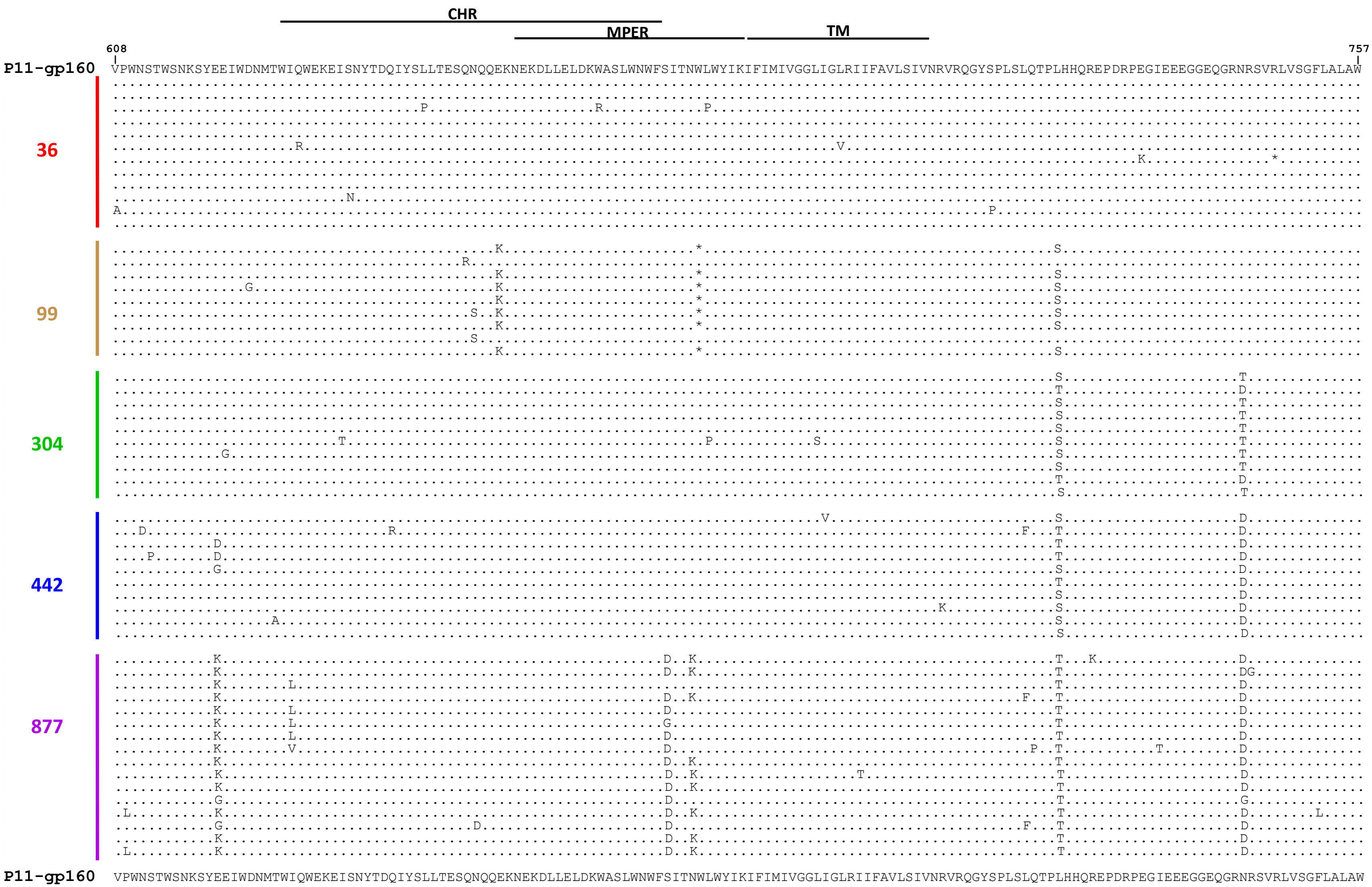
CHR
MPER
TM
608
757
P11-gp160
VPWNSTWSNKSYEEIWDNMTWIQWEKEISNYTDQIYSLLTESQNQQEKNEKDLLELDKWASLWNWFSITNWLWYIKIFIMIVGGLIGLRIIFAVLSIVNRVRQGYSPLSLQTPLHHQREPDRPEGIEEEGGEQGRNRSVRLVSGFLALAW
36
99
304
442
877
P11-gp160
VPWNSTWSNKSYEEIWDNMTWIQWEKEISNYTDQIYSLLTESQNQQEKNEKDLLELDKWASLWNWFSITNWLWYIKIFIMIVGGLIGLRIIFAVLSIVNRVRQGYSPLSLQTPLHHQREPDRPEGIEEEGGEQGRNRSVRLVSGFLALAW

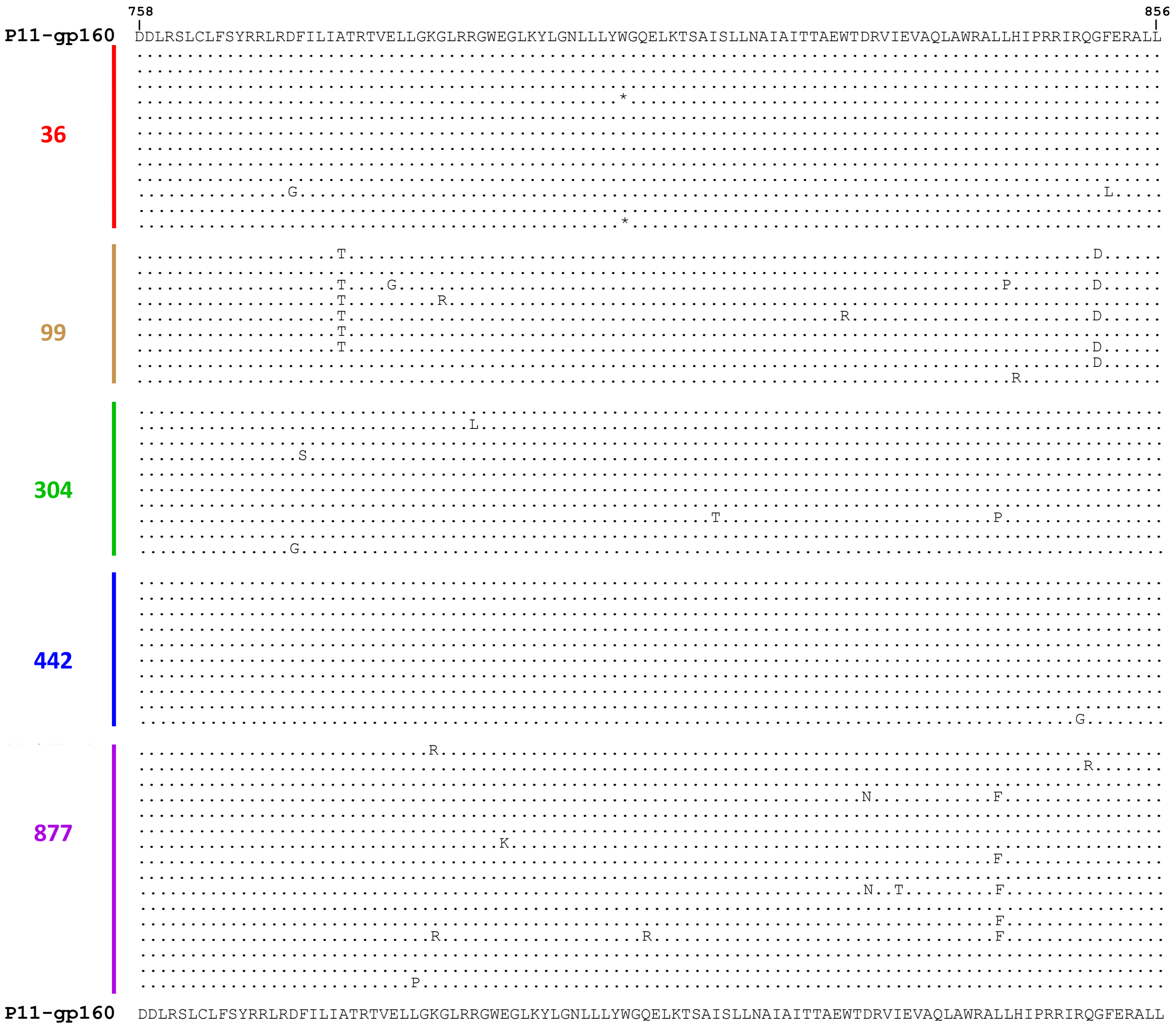
758
856
P11-gp160 DDLRSLCLFSYRRLRDFILIATRTVELLGKGLRRGWEGLKYLGNLLLYWGQELKTSAISLLNAIAITTAEWTDRVIEVAQLAWRALLHIPRRIRQGFERALL
36
99
304
442
877
P11-gp160 DDLRSLCLFSYRRLRDFILIATRTVELLGKGLRRGWEGLKYLGNLLLYWGQELKTSAISLLNAIAITTAEWTDRVIEVAQLAWRALLHIPRRIRQGFERALL

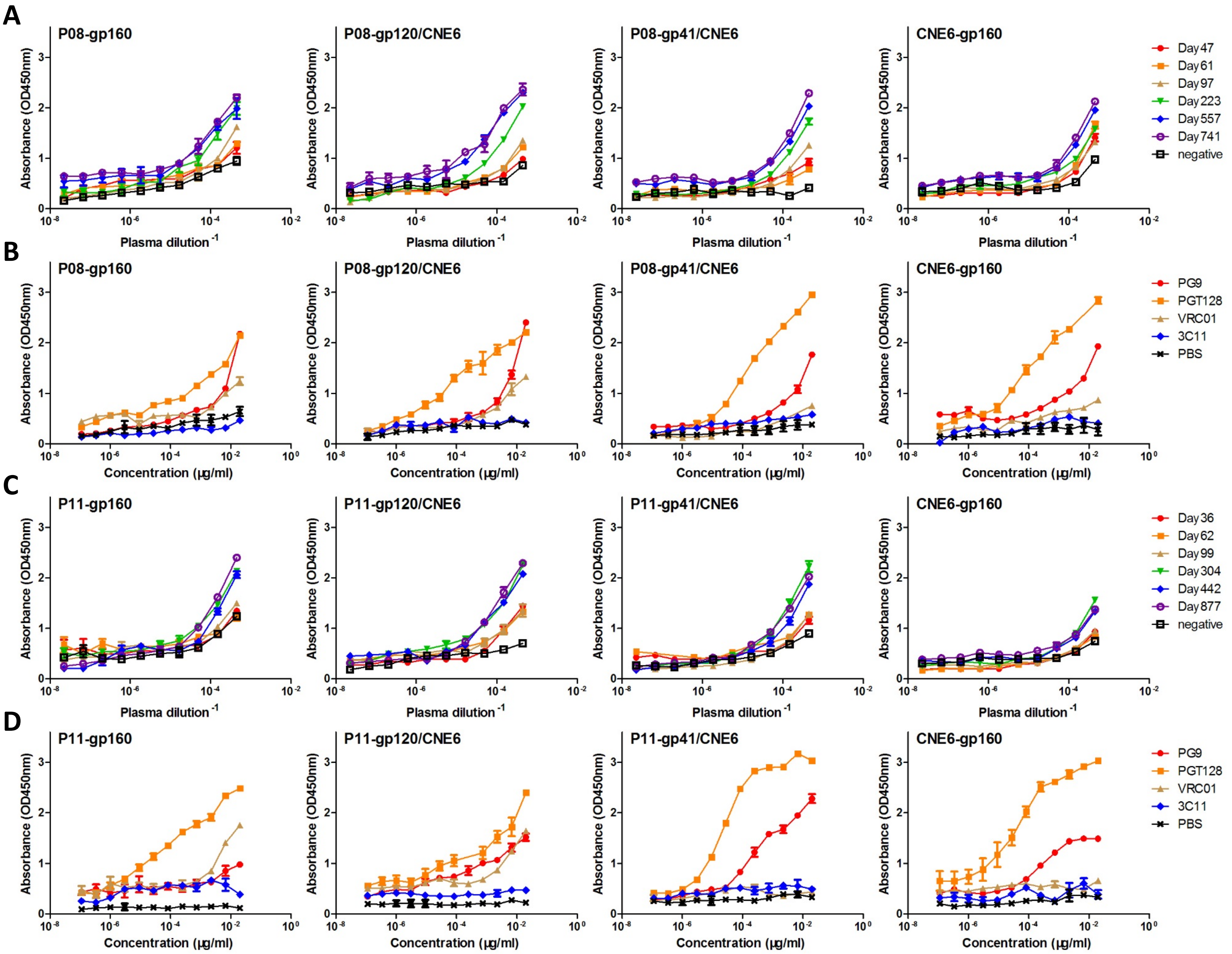
A
P08-gp160
P08-gp120/CNE6
P08-gp41/CNE6
CNE6-gp160
Absorbance (OD450nm)
Plasma dilution -1
Day47
Day61
Day97
Day223
Day557
Day741
negative
B
P08-gp160
P08-gp120/CNE6
P08-gp41/CNE6
CNE6-gp160
Concentration (μg/ml)
PG9
PGT128
VRC01
3C11
PBS
C
P11-gp160
P11-gp120/CNE6
P11-gp41/CNE6
CNE6-gp160
Plasma dilution -1
Day36
Day62
Day99
Day304
Day442
Day877
negative
D
P11-gp160
P11-gp120/CNE6
P11-gp41/CNE6
CNE6-gp160
Concentration (μg/ml)
PG9
PGT128
VRC01
3C11
PBS

Figure S3

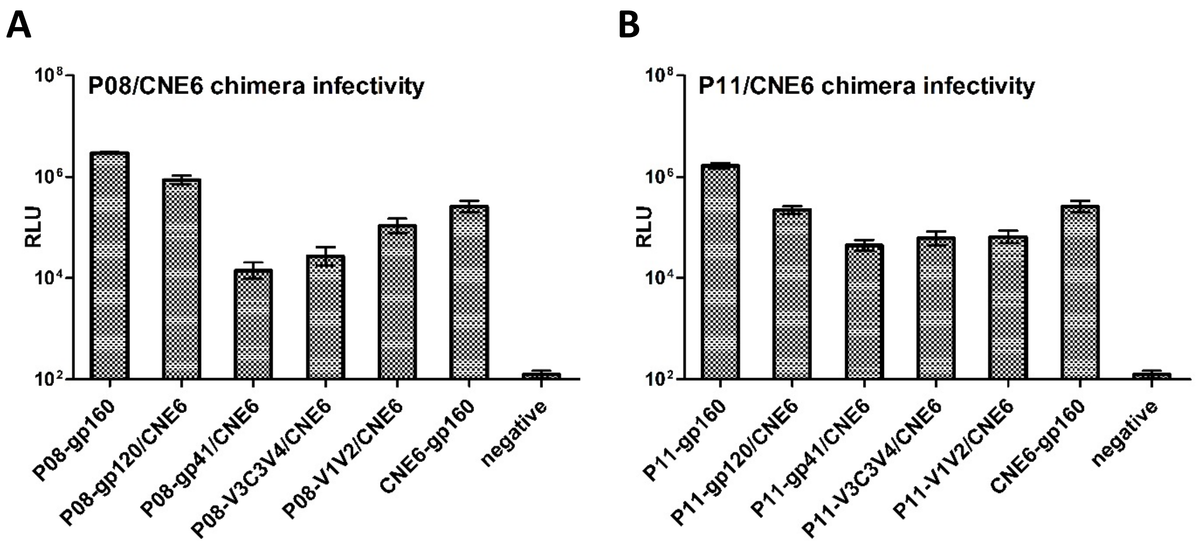
A
P08/CNE6 chimera infectivity
RLU
10^8
10^6
10^4
10^2
P08-gp160
P08-gp120/CNE6
P08-gp41/CNE6
P08-V3C3V4/CNE6
P08-V1V2/CNE6
CNE6-gp160
negative
B
P11/CNE6 chimera infectivity
RLU
10^8
10^6
10^4
10^2
P11-gp160
P11-gp120/CNE6
P11-gp41/CNE6
P11-V3C3V4/CNE6
P11-V1V2/CNE6
CNE6-gp160
negative

Figure S4
